# The m6A demethylase FTO suppresses glioma proliferation by regulating the EREG/PI3K/Akt signaling pathway

**DOI:** 10.3389/fcell.2025.1667990

**Published:** 2025-09-03

**Authors:** Yong Zhai, Caili Li, Lihui Cao, Shen Zhang, Xiao Liu, Junwei Ren, Yue Liu

**Affiliations:** ^1^ Emergency Department, Brain-Computer Interface and Neural Repair Laboratory, Xiangyang No. 1 People’s Hospital, Hubei University of Medicine, Xiangyang, Hubei, China; ^2^ Department of Health Management Center, Brain-Computer Interface and Neural Repair Laboratory, Xiangyang No. 1 People’s Hospital, Hubei University of Medicine, Xiangyang, Hubei, China; ^3^ Department of Neurosurgery, Brain-Computer Interface and Neural Repair Laboratory, Xiangyang No. 1 People’s Hospital, Hubei University of Medicine, Xiangyang, Hubei, China

**Keywords:** glioma, N6-methyladenosine, FTO, tumor suppressor, cell cycle, PI3K/Akt pathway

## Abstract

**Background:**

Glioma, the most prevalent primary intracranial tumor, is characterized by aggressive proliferation and formidable treatment challenges. The N6-methyladenosine (m6A) demethylase, Fat mass and obesity-associated protein (FTO), is a critical regulator of gene expression, but its precise role in glioma remains controversial. This study aimed to elucidate the function and underlying molecular mechanisms of FTO in glioma progression.

**Methods:**

We integrated bioinformatic analysis of 1,027 glioma patients from public cohorts (TCGA and CGGA) with a comprehensive experimental approach. *In vitro* studies in U251 and U87MG glioma cells involved gain- and loss-of-function assays to assess proliferation, colony formation, and cell cycle progression. Mechanistic investigations included Western blotting, qRT-PCR, and mRNA stability assays. An *in vivo* subcutaneous xenograft model was used to validate the tumor-suppressive role of FTO.

**Results:**

Our analysis revealed that lower FTO expression is significantly associated with higher tumor grade and poorer overall survival in glioma patients. Functionally, FTO overexpression inhibited proliferation and induced G1 phase cell cycle arrest, whereas FTO knockdown enhanced these malignant phenotypes. Mechanistically, we identified Epiregulin (EREG) as a key downstream target of FTO. Loss of FTO increased global m6A levels and enhanced EREG mRNA stability, leading to its upregulation. This, in turn, activated the PI3K/Akt signaling pathway, evidenced by increased phosphorylation of PI3K and Akt and subsequent downregulation of p53 and p21. The *in vivo* model confirmed that FTO overexpression suppressed tumor growth, while its knockdown accelerated it.

**Conclusion:**

Our findings establish FTO as a tumor suppressor in glioma. It inhibits proliferation by destabilizing EREG mRNA in an m6A-dependent manner, thereby inactivating the PI3K/Akt signaling cascade. These results highlight FTO as a potential prognostic biomarker and a promising therapeutic target for glioma.

## 1 Introduction

Glioma, originating from neuroglial cells, is the most common and aggressive primary intracranial tumor, with an annual incidence of 5–8 per 100,000 people (Ostrom et al., 2022). Despite multimodal treatment strategies including surgery, radiotherapy, and chemotherapy, the prognosis for patients, particularly those with high-grade gliomas like glioblastoma (GBM), remains dismal due to relentless cell proliferation, cell cycle dysregulation, and high rates of recurrence (Hussain et al., 2023; Wesseling and Capper, 2018). A key challenge is profound intra- and inter-tumoral heterogeneity and the development of resistance to standard-of-care treatments like temozolomide (TMZ) (Sherman et al., 2024). According to the 2021 World Health Organization (WHO) Classification of Tumors of the Central Nervous System, molecular markers are now integral to glioma diagnosis and prognosis (Louis et al., 2021). Therefore, elucidating the molecular drivers of glioma’s malignant progression is crucial for developing novel and effective therapeutic interventions.

N6-methyladenosine (m6A) is the most abundant internal modification on eukaryotic messenger RNA (mRNA), dynamically regulated by a complex interplay of “writer” (methyltransferases), “eraser” (demethylases), and “reader” (binding proteins) enzymes (An and Duan, 2022; Zaccara et al., 2019). This regulatory network, comprising writers like the METTL3-METTL14 complex, erasers such as FTO and ALKBH5, and readers like the YTH-domain family proteins, provides a sophisticated layer of post-transcriptional control (Shi et al., 2019). This reversible epigenetic mark governs nearly every aspect of RNA metabolism, including splicing, stability, translation, and nuclear export (Jiang et al., 2021). Growing evidence indicates that dysregulation of the m6A machinery is deeply implicated in the pathogenesis of various cancers by modulating the expression of key oncogenes and tumor suppressors, thereby influencing processes such as tumor initiation, immune evasion, and metabolic reprogramming (Sun et al., 2019; Liu et al., 2025).

The Fat Mass and Obesity-Associated (FTO) protein, first identified for its association with obesity, was later discovered as the first RNA m6A demethylase (Jia et al., 2011; Gerken et al., 2007). FTO plays a multifaceted role in tumorigenesis, with its function appearing to be context-dependent. It has been reported as an oncogene in acute myeloid leukemia and a tumor suppressor in other cancers (Li et al., 2022; Zhao et al., 2014). For instance, FTO has been shown to promote tumorigenesis in non-small cell lung cancer and hepatocellular carcinoma by targeting key oncogenes or metabolic pathways (Gao et al., 2023; Li et al., 2019). In glioma, the role of FTO remains debated. Some studies have shown that FTO expression is negatively correlated with glioma malignancy and that patients with lower FTO levels have a poorer prognosis (Chai et al., 2019; Tao et al., 2020). Others suggest that inhibiting FTO activity could be a viable therapeutic strategy (Huang et al., 2019). These conflicting reports underscore the need for a comprehensive investigation into FTO’s precise function and mechanism in glioma.

Given the consistent clinical observation of reduced FTO expression in high-grade gliomas, we hypothesized that FTO functions as a tumor suppressor. In this study, we first leveraged large-scale public datasets (TCGA and CGGA) to confirm the correlation between low FTO expression and adverse clinical features in a cohort of 1,027 glioma patients. We then employed *in vitro* gain- and loss-of-function studies in U251 and U87MG glioma cell lines, coupled with an *in vivo* xenograft model, to systematically investigate the biological role of FTO. Our findings demonstrate that FTO suppresses glioma proliferation and cell cycle progression by regulating the stability of Epiregulin (EREG) mRNA and subsequently inactivating the PI3K/Akt signaling pathway, thereby establishing its role as a key tumor suppressor in glioma.

## 2 Materials and methods

### 2.1 Public database analysis and patient samples

This study utilized a retrospective cohort design for bioinformatic analysis. Publicly available RNA-sequencing data and corresponding clinical information for 1,027 glioma patients were downloaded from The Cancer Genome Atlas (TCGA, https://portal.gdc.cancer.gov/) and the Chinese Glioma Genome Atlas (CGGA, http://www.cgga.org.cn/) databases (Zhao et al., 2021; Brennan et al., 2013). Key variables extracted included FTO expression levels, overall survival (OS), WHO grade, histological type, IDH mutation status, and 1p/19q co-deletion status. The study protocol for using public data was exempt from institutional review board approval.

Additionally, a total of 25 fresh-frozen tissue samples, comprising 8 low-grade gliomas (2 Grade I, 6 Grade II), 14 high-grade gliomas (6 Grade III, 8 Grade IV), and 3 normal brain tissues (obtained during surgical decompression for trauma), were collected from the Department of Neurosurgery of Xiangyang No. 1 People’s Hospital, Hubei University of Medicine, between October 2021 and June 2023. All participants provided written informed consent. The study was approved by the Ethics Committee on Scientific Research of Xiangyang No. 1 People’s Hospital (Approval No. 2021KYLX05) and conducted in accordance with the Declaration of Helsinki.

### 2.2 Cell culture and reagents

Human glioma cell lines U251 (Cat# CL-0237) and U87MG (Cat# CL-0238), and the human microglial cell line HMC3 (Cat# CL-0620), were purchased from Procell Life Science & Technology Co., Ltd. (Wuhan, China). Cells were cultured in Dulbecco’s Modified Eagle Medium (DMEM, Cat# PM150210; Procell) supplemented with 10% Fetal Bovine Serum (FBS, Cat# SA211.01; CellMax) and 1% Penicillin-Streptomycin solution (Procell). All cells were maintained in a humidified incubator at 37 °C with 5% CO_2_. *Mycoplasma* contamination was routinely tested using a PCR-based kit. The PI3K inhibitor LY294002 (Selleck Chemicals, United States of America), FTO inhibitor FB23-2 (Huang et al., 2019), and transcription inhibitor Actinomycin D (Sigma-Aldrich, United States) were used as indicated.

### 2.3 Functional enrichment analysis

Patients from the TCGA and CGGA cohorts were stratified into high- and low-FTO expression groups based on the median expression value. Differentially expressed genes (DEGs) were identified using the DESeq2 R package with criteria of |log2(Fold Change)| > 1 and adjusted p-value <0.05. The identified DEGs were uploaded to the Database for Annotation, Visualization, and Integrated Discovery (DAVID, v6.8, https://david.ncifcrf.gov/) for Gene Ontology (GO) and Kyoto Encyclopedia of Genes and Genomes (KEGG) pathway enrichment analyses (Sherman et al., 2022). The top enriched terms (p < 0.05) are presented (Supplementary Tables S1–S4).

### 2.4 Lentivirus transduction and stable cell line generation

Lentiviral vectors for FTO overexpression (oeFTO) were constructed by cloning the human full-length FTO cDNA sequence into the GV492 vector. A corresponding empty vector served as a negative control (oeNC). For FTO knockdown, two short hairpin RNA (shRNA) sequences targeting FTO (shFTO-1, shFTO-2) and a non-targeting scramble control (shNC) were cloned into the pLKD vector. All lentiviral constructs were synthesized and packaged by GenePharma (Shanghai, China). Target sequences are listed in Supplementary Table S5.

U251 and U87MG cells were seeded in 6-well plates and transduced with lentiviruses at approximately 50% confluency in the presence of polybrene (8 μg/mL). After 48 h, the medium was replaced with fresh medium containing puromycin (4 μg/mL; Thermo Fisher Scientific) for 2 weeks to select for stably transduced cells. Transfection efficiency was confirmed by qRT-PCR and Western blot.

### 2.5 Total m6A quantification assay

Total RNA was extracted from cells using TRIzol reagent (Cat# 10606ES60; YEASEN). The global m6A level in 200 ng of total RNA was measured using the EpiQuik m6A RNA Methylation Quantification Kit (Colorimetric) (Cat# P-9005-48; EpiGentek) according to the manufacturer’s protocol (Shi et al., 2021). Absorbance was read at 450 nm, and the relative m6A level was calculated based on a standard curve.

### 2.6 Cell proliferation and colony formation assays

For the 5-ethynyl-2′-deoxyuridine (EdU) assay, cells were seeded in 24-well plates (5 × 10^4^ cells/well). After 24 h, cell proliferation was assessed using a BeyoClick™ EdU Cell Proliferation Kit with Alexa Fluor 488 (Cat# C0071S; Beyotime) following the manufacturer’s instructions. EdU-positive (green) and DAPI-stained (blue) nuclei were imaged using a fluorescence microscope, and the percentage of EdU-positive cells was quantified from at least five random fields.

For the colony formation assay, 300 cells per well were seeded into 6-well plates and cultured for 10–14 days until visible colonies formed. The colonies were fixed with 4% paraformaldehyde and stained with 0.1% crystal violet solution. Colonies containing more than 50 cells were counted.

### 2.7 Cell cycle analysis by flow cytometry

Cells were harvested, washed with ice-cold PBS, and fixed in 70% ethanol at −20 °C overnight. After fixation, cells were washed again and resuspended in PBS containing propidium iodide (PI, 50 μg/mL) and RNase A (100 μg/mL) from a Cell Cycle Staining Kit (Cat# BL114A; Biosharp). After incubation for 30 min in the dark, the DNA content was analyzed using a flow cytometer (Beckman Coulter). The percentages of cells in G1, S, and G2/M phases were quantified using ModFit LT software.

### 2.8 Quantitative real-time PCR (qRT-PCR)

Total RNA was isolated using TRIzol, and 1 µg was reverse-transcribed into cDNA using the RevertAid First Strand cDNA Synthesis Kit (Takara, Japan). qRT-PCR was performed on a Bio-Rad CFX96 Real-Time PCR System using SYBR Green Master Mix (Takara, Japan). The relative expression of target genes was calculated using the 2^−ΔΔCT^ method, with GAPDH serving as the endogenous control. Primer sequences are provided in Supplementary Table S5.

### 2.9 Western blot analysis

Total protein was extracted using RIPA lysis buffer (Cat# BL651A; Biosharp) supplemented with protease and phosphatase inhibitor cocktails. Protein concentrations were determined using a BCA assay kit. Equal amounts of protein (20–30 µg) were separated by SDS-PAGE and transferred to PVDF membranes. The membranes were blocked with 5% non-fat milk and incubated overnight at 4 °C with the following primary antibodies: FTO (1:1000, Cat# R24361; ZENBIO), EREG (1:1000, Cat# 12048; Cell Signaling Technology), PI3K (1:1000, Cat# 60225-1-Ig; Proteintech), p-PI3K (1:1000, Cat# ab38449; Abcam), Akt (1:2000, Cat# 60203-2-Ig; Proteintech), p-Akt (Ser473) (1:2000, Cat# 80455-1-RR; Proteintech), p53 (1:1000, Cat# abs130605; Absin), p21 (1:1000, Cat# YT3497; Immunoway), and GAPDH (1:10000, Cat# ANT324s; AntGene). After incubation with appropriate HRP-conjugated secondary antibodies (1:5000; Proteintech), protein bands were visualized using an enhanced chemiluminescence (ECL) kit (Beyotime, China) and quantified using ImageJ software.

### 2.10 Immunohistochemistry (IHC)

Paraffin-embedded tissue sections (5 μm) from clinical samples and subcutaneous xenograft tumors were deparaffinized and rehydrated. Antigen retrieval was performed by heating in citrate buffer (pH 6.0). The sections were then incubated with primary antibodies against FTO, EREG, or p53 overnight at 4 °C, followed by incubation with an HRP-conjugated secondary antibody. Staining was developed with DAB chromogen and counterstained with hematoxylin. Staining intensity was semi-quantitatively scored based on the proportion of positive cells and staining intensity, calculated as the integrated optical density (IOD) per area using Image-Pro Plus software.

### 2.11 Animal studies

All animal experiments were conducted in strict accordance with the ARRIVE guidelines and were approved by the Animal Use and Care Committee of Xiangyang No. 1 People’s Hospital (Approval No. 2021KYLX05). Male BALB/c nude mice (4–6 weeks old) were purchased from Hunan Silaikejingda Experimental Animal Co., Ltd. (License No. SCXK (Hunan) 2019-0004) and housed under specific pathogen-free conditions with a 12-h light/dark cycle and *ad libitum* access to food and water.

After 1 week of acclimatization, a subcutaneous xenograft model was established. A total of 28 mice were randomly allocated into four groups (*n* = 7 per group) using a simple randomization method. Prior to injection, mice were briefly anesthetized using inhaled isoflurane (2%–3%). Subsequently, U251 cells stably expressing oeNC, oeFTO, shNC, or shFTO-1 (5 × 10^6^ cells in 100 µL PBS) were injected subcutaneously into the left flank of each mouse. Tumor growth was monitored every 3 days by measuring the length (L) and width (W) with calipers. Tumor volume was calculated using the formula: Volume = (L × W^2^)/2. After 28 days, mice were euthanized by CO_2_ inhalation using a gradual displacement rate of 20% of the chamber volume per minute, followed by cervical dislocation to ensure death, and the tumors were excised, weighed, and processed for IHC analysis. No animals were excluded from the analysis.

### 2.12 Statistical analysis

All experiments were performed in triplicate unless otherwise specified. Data are presented as mean ± standard deviation (SD). Statistical analyses were performed using GraphPad Prism 8.0 (GraphPad Software, United States) and R software (v4.1.2). Comparisons between two groups were analyzed using an unpaired two-tailed Student’s t-test. For comparisons among three or more groups, one-way analysis of variance (ANOVA) followed by Tukey’s *post hoc* test was used. Kaplan-Meier survival curves were generated, and the log-rank test was used to compare survival differences. A p-value <0.05 was considered statistically significant. Significance levels are denoted as *p < 0.05, **p < 0.01, and ***p < 0.001.

## 3 Results

### 3.1 Low FTO expression correlates with malignant features and poor prognosis in glioma

**FIGURE 1:**
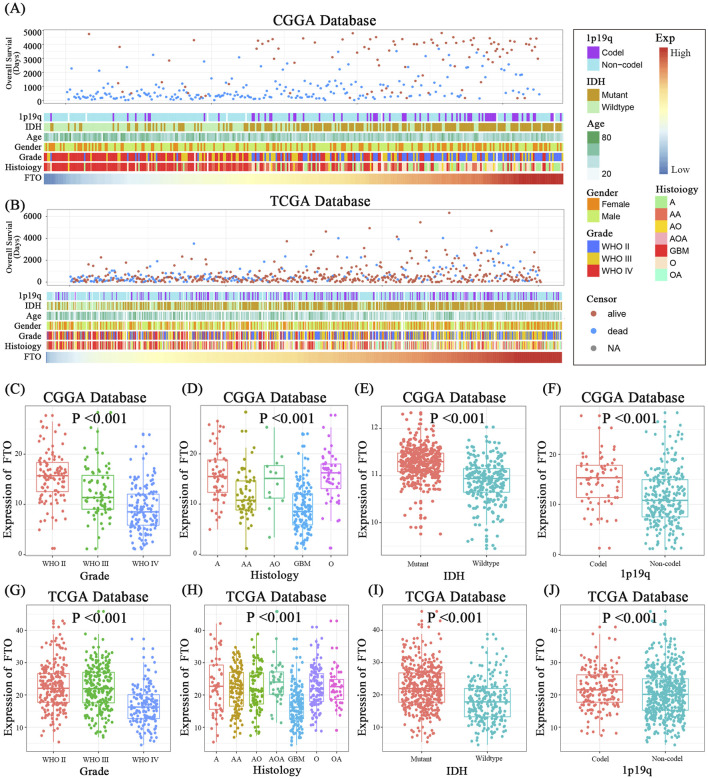
Low FTO expression is associated with clinicopathological features of high-grade glioma. **(A,B)** Heatmaps displaying the correlation between FTO expression and key clinical characteristics (overall survival, age, gender, WHO grade, histology, IDH status, 1p/19q status) in patients from the CGGA **(A)** and TCGA **(B)** databases. **(C,G)** Box plots showing significantly lower FTO expression in high-grade gliomas (WHO grades III-IV) compared to low-grade gliomas (WHO I-II) in both cohorts. **(D,H)** FTO expression is significantly reduced in various histological subtypes, particularly GBM, in both cohorts. **(E,I)** FTO expression is lower in IDH wild-type gliomas compared to IDH mutant gliomas. **(F,J)** FTO expression is lower in gliomas with non-codel 1p/19q status compared to those with co-deletion. Statistical significance was assessed using one-way ANOVA for multi-group comparisons **(C,D,G,H)** and unpaired two-tailed t-test for two-group comparisons **(E,F,I,J)**. Data are shown as mean ± SD. ***p < 0.001.

To investigate the clinical relevance of FTO in glioma, we analyzed transcriptomic and clinical data from the CGGA and TCGA cohorts. Heatmaps of patient characteristics stratified by FTO expression revealed that low FTO levels were associated with adverse features, including higher WHO grade, wild-type IDH status, and non-codel 1p/19q status (Figures 1A,B). Quantitative analysis confirmed that FTO expression was significantly lower in high-grade gliomas (WHO grades III-IV) compared to low-grade gliomas (WHO grades I-II) (Figures 1C,G). FTO levels were also markedly reduced in the most aggressive histological subtype, glioblastoma (GBM), and in patients with wild-type IDH and non-codel 1p/19q status (Figures 1D–F,H–J).

**FIGURE 2:**
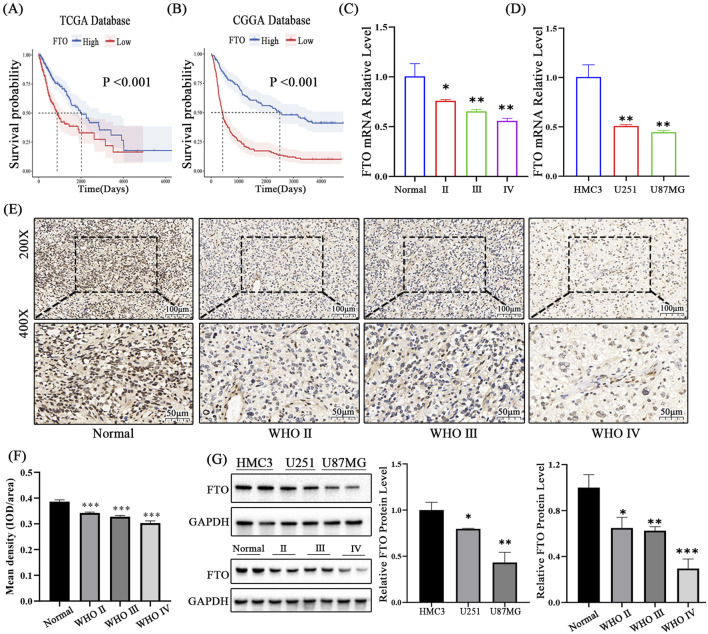
Low FTO expression predicts poor prognosis and is confirmed in clinical samples and cell lines. **(A,B)** Kaplan-Meier survival curves demonstrating that low FTO expression is significantly associated with shorter overall survival in both the TCGA **(A)** and CGGA **(B)** cohorts (log-rank test). **(C,D)** qRT-PCR analysis of FTO mRNA levels in clinical glioma tissue samples of different WHO grades **(C)** and in glioma cell lines (U251, U87MG) compared to normal microglial cells (HMC3) **(D)**. **(E)** Representative IHC images of FTO protein expression in normal brain tissue and glioma tissues of increasing WHO grades. Scale bars: 100 μm (200×), 50 μm (400×). **(F)** Quantification of IHC staining intensity (IOD/area). **(G)** Western blot analysis and quantification of FTO protein levels in clinical samples and cell lines. GAPDH was used as a loading control. Data are shown as mean ± SD from at least three independent experiments. *p < 0.05, **p < 0.01, ***p < 0.001.

Kaplan-Meier survival analysis demonstrated that patients with low FTO expression had significantly shorter overall survival (OS) in both the CGGA and TCGA cohorts (Figures 2A,B). To validate these bioinformatic findings, we examined FTO expression in our own clinical samples. IHC and qRT-PCR analyses showed a progressive decrease in FTO protein and mRNA levels with increasing glioma grade, consistent with the database results (Figures 2C,E,F). Furthermore, FTO expression was significantly lower in U251 and U87MG glioma cell lines compared to the HMC3 normal microglial cell line (Figures 2D,G). Collectively, these data strongly indicate that FTO is downregulated in malignant glioma and that its low expression is a marker of poor prognosis, suggesting a tumor-suppressive role.

### 3.2 Biofunctional analysis reveals FTO’s association with proliferation and the PI3K/Akt pathway

**FIGURE 3:**
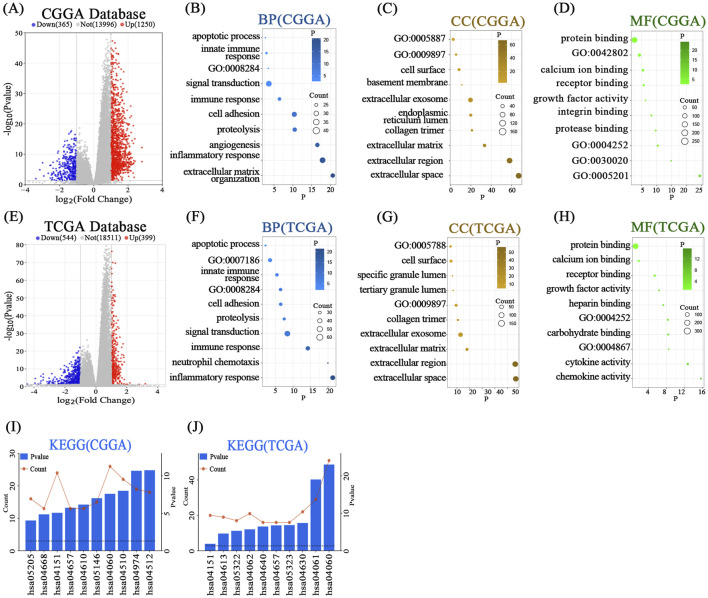
Functional enrichment analysis links FTO to cell proliferation and the PI3K/Akt pathway. **(A,E)** Volcano plots showing differentially expressed genes (DEGs) between high- and low-FTO expression groups in the CGGA **(A)** and TCGA **(E)** cohorts. **(B–D,F–H)** GO enrichment analysis of DEGs for Biological Processes (BP), Cellular Components (CC), and Molecular Functions (MF) in the CGGA **(B–D)** and TCGA **(F–H)** cohorts. **(I,J)** KEGG pathway enrichment analysis of DEGs in the CGGA **(I)** and TCGA **(J)** cohorts. The PI3K-Akt signaling pathway (hsa04151) is highlighted as a significantly enriched pathway.

To explore the molecular functions regulated by FTO, we performed GO and KEGG pathway analyses on the DEGs between high- and low-FTO expression groups. In both the CGGA and TCGA datasets, GO analysis revealed that genes co-expressed with FTO were significantly enriched in biological processes related to cell proliferation, apoptosis, and immune response (Figures 3A–H). Notably, KEGG pathway analysis consistently highlighted the PI3K/Akt signaling pathway (hsa04151) as one of the most significantly enriched pathways associated with FTO expression (Figures 3I,J). These bioinformatic predictions suggest that FTO may exert its tumor-suppressive effects by modulating cell proliferation via the PI3K/Akt pathway.

### 3.3 FTO overexpression suppresses glioma cell proliferation and induces cell cycle arrest

**FIGURE 4:**
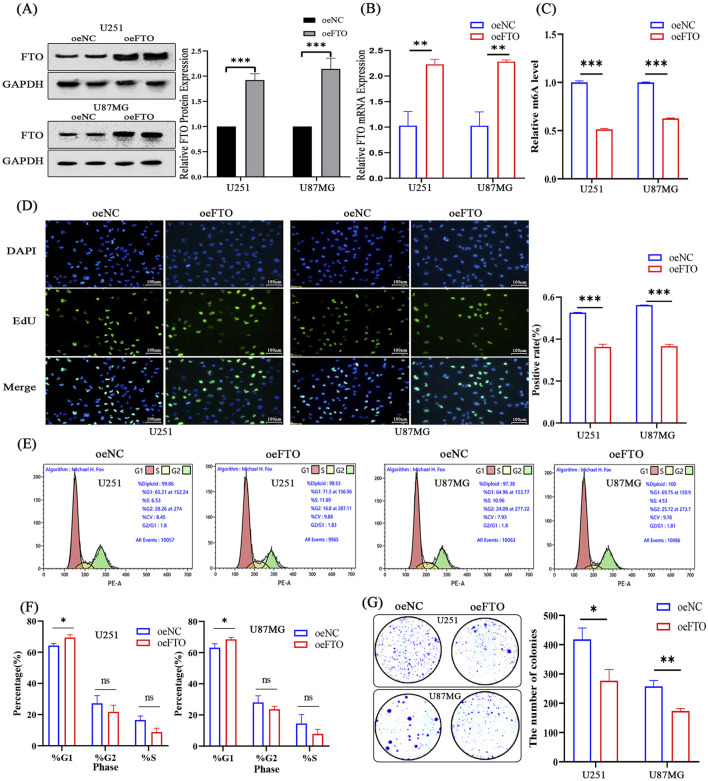
Overexpression of FTO suppresses glioma cell proliferation and induces G1 phase cell cycle arrest. **(A,B)** Western blot **(A)** and qRT-PCR **(B)** confirming successful FTO overexpression (oeFTO) in U251 and U87MG cells compared to negative control (oeNC). **(C)** FTO overexpression significantly decreases global m6A levels in glioma cells. **(D)** Representative images and quantification of EdU incorporation assays showing reduced proliferation in oeFTO cells. Scale bar: 100 µm. **(E,F)** Representative flow cytometry plots **(E)** and quantification **(F)** of cell cycle distribution, showing G1 phase arrest in oeFTO cells. **(G)** Quantification of colony formation assays demonstrating that FTO overexpression inhibits the clonogenic capacity of glioma cells. Data are shown as mean ± SD from three independent experiments. *p < 0.05, **p < 0.01, ***p < 0.001. ns, not significant.

To directly test the tumor-suppressive function of FTO, we generated U251 and U87MG cell lines stably overexpressing FTO (oeFTO). Successful overexpression was confirmed at both mRNA and protein levels (Figures 4A,B). As expected, FTO overexpression significantly decreased the global m6A RNA levels, confirming its demethylase activity (Figure 4C). Functional assays showed that the oeFTO group had a significantly lower proportion of EdU-positive cells compared to the control (oeNC) group, indicating inhibited proliferation (Figure 4D). Similarly, FTO overexpression markedly reduced the number and size of colonies in the colony formation assay (Figure 4G). Flow cytometry analysis revealed that FTO overexpression caused a significant accumulation of cells in the G1 phase, with a corresponding decrease in the S phase population, indicating G1 phase arrest (Figures 4E,F). These results demonstrate that FTO suppresses the malignant growth of glioma cells *in vitro*.

### 3.4 FTO knockdown promotes glioma cell proliferation and cell cycle progression

**FIGURE 5:**
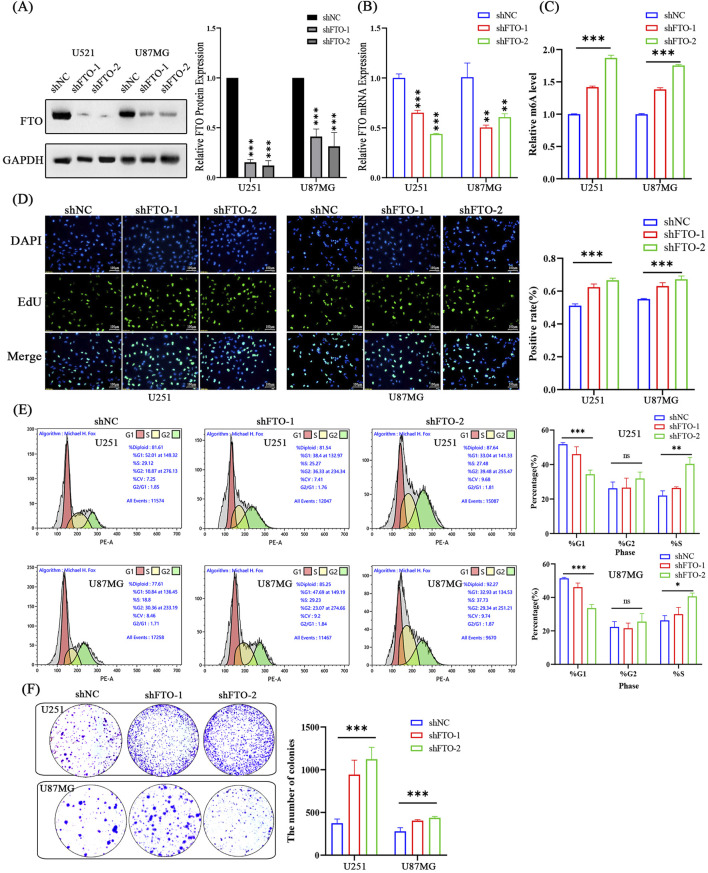
Knockdown of FTO promotes glioma cell proliferation and accelerates cell cycle progression. **(A,B)** Western blot **(A)** and qRT-PCR **(B)** confirming efficient FTO knockdown using two shRNAs (shFTO-1, shFTO-2) in U251 and U87MG cells compared to scramble control (shNC). **(C)** FTO knockdown significantly increases global m6A levels. **(D)** Representative images and quantification of EdU assays showing enhanced proliferation in FTO-knockdown cells. Scale bar: 100 µm. **(E)** Representative flow cytometry plots and quantification showing accelerated G1/S transition in FTO-knockdown cells. **(F)** Quantification of colony formation assays demonstrating that FTO knockdown enhances the clonogenic capacity of glioma cells. Data are shown as mean ± SD from three independent experiments. *p < 0.05, **p < 0.01, ***p < 0.001.

Conversely, to mimic the low FTO expression observed in high-grade glioma, we knocked down FTO using two independent shRNAs (shFTO-1, shFTO-2). Both shRNAs effectively reduced FTO mRNA and protein levels (Figures 5A,B) and led to a significant increase in global m6A levels (Figure 5C). FTO knockdown robustly enhanced cell proliferation, as shown by an increased percentage of EdU-positive cells (Figure 5D) and a greater number of colonies formed (Figure 5F). Cell cycle analysis showed that FTO knockdown resulted in a decreased G1 phase population and an increased S phase population, indicating accelerated cell cycle progression (Figure 5E). These findings corroborate our overexpression results and confirm that loss of FTO promotes the proliferative capacity of glioma cells.

### 3.5 FTO suppresses glioma growth *in vivo*

**FIGURE 6:**
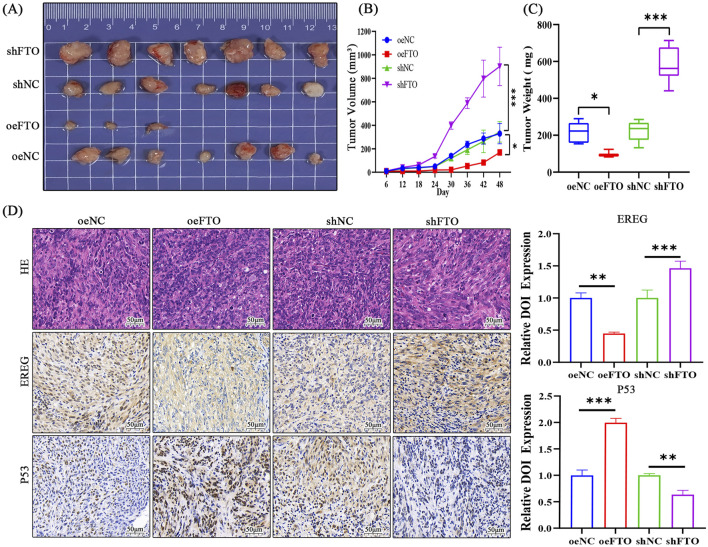
FTO suppresses glioma tumor growth in a subcutaneous xenograft model. **(A)** Images of tumors excised from nude mice (*n* = 7 per group) 28 days after subcutaneous injection of U251 cells stably expressing oeNC, oeFTO, shNC, or shFTO-1. **(B)** Tumor volume growth curves measured every 3 days. **(C)** Box plot of final tumor weights at the time of sacrifice. **(D)** Representative H&E staining and IHC staining for EREG and p53 in sections from xenograft tumors. FTO overexpression decreased EREG and increased p53 staining, while FTO knockdown showed the opposite effects. Scale bar: 50 µm. Data are shown as mean ± SD. *p < 0.05, ***p < 0.001.

To validate the tumor-suppressive role of FTO *in vivo*, we established a subcutaneous xenograft model using U251 cells with stable FTO overexpression (oeFTO) or knockdown (shFTO-1). The results were striking: tumors in the oeFTO group grew significantly slower and were substantially smaller and lighter at the end of the experiment compared to the oeNC group (Figures 6A–C). In contrast, tumors in the shFTO group grew much faster and were significantly larger and heavier than those in the shNC group. IHC analysis of the excised tumors revealed that oeFTO tumors had lower EREG expression and higher p53 expression, whereas shFTO tumors showed the opposite trend (Figure 6D). These *in vivo* data provide compelling evidence that FTO acts as a potent suppressor of glioma tumor growth.

### 3.6 FTO regulates EREG stability via its m6A demethylase activity

**FIGURE 7:**
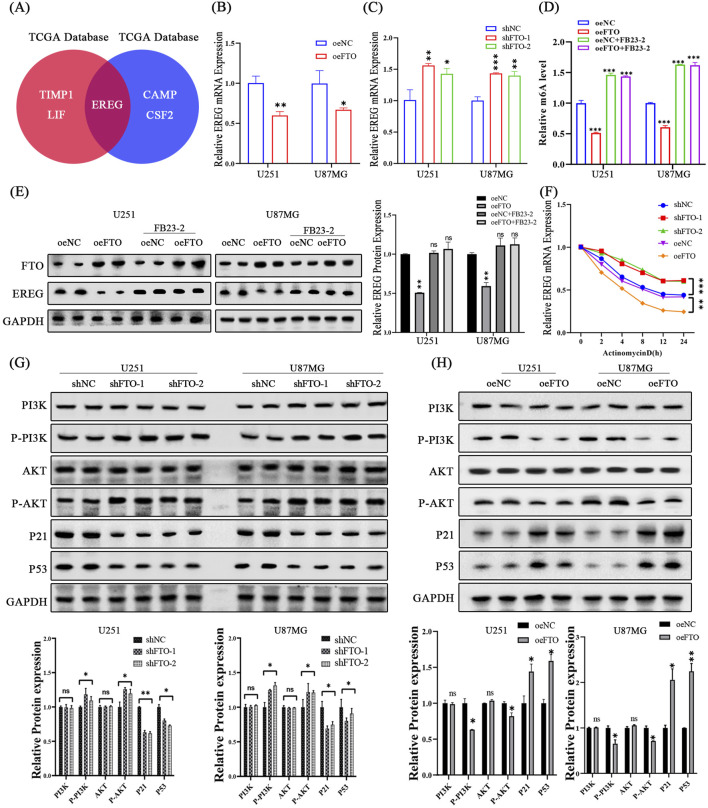
FTO regulates the EREG/PI3K/Akt pathway in an m6A-dependent manner. **(A)** Venn diagram showing EREG as a key overlapping gene associated with cell proliferation regulated by FTO. **(B,C)** qRT-PCR analysis showing that FTO overexpression decreases EREG mRNA levels **(B)**, while FTO knockdown increases them **(C)**. **(D,E)** The FTO inhibitor FB23-2 reverses the FTO-overexpression-induced decrease in global m6A levels **(D)** and restores EREG protein expression **(E)**. **(F)** mRNA stability assay using Actinomycin D shows that FTO overexpression shortens the half-life of EREG mRNA, while FTO knockdown prolongs it. **(G,H)** Western blot analysis and quantification showing that FTO knockdown activates the PI3K/Akt pathway (increased p-PI3K, p-Akt) and downregulates p53 and p21 **(G)**, while FTO overexpression has the opposite effects **(H)**. Data are shown as mean ± SD from three independent experiments. *p < 0.05, **p < 0.01, ***p < 0.001.

To identify the downstream effector of FTO, we searched for DEGs associated with cell proliferation and found that Epiregulin (EREG), a member of the epidermal growth factor family, was a top candidate consistently regulated by FTO in both public datasets (Figure 7A). We confirmed this at the molecular level: FTO overexpression decreased EREG mRNA and protein levels, while FTO knockdown increased them (Figures 7B,C,G,H). To determine if this regulation was dependent on FTO’s demethylase activity, we treated oeFTO cells with the FTO inhibitor FB23-2. The inhibitor reversed the FTO-induced decrease in global m6A levels and restored EREG protein expression to control levels (Figures 7D,E). This suggests FTO regulates EREG in a demethylase-dependent manner.

Next, we investigated whether FTO affects EREG mRNA stability. Cells were treated with the transcription inhibitor Actinomycin D, and EREG mRNA levels were measured over time. The half-life of EREG mRNA was significantly shorter in FTO-overexpressing cells and markedly longer in FTO-knockdown cells compared to their respective controls (Figure 7F). This indicates that FTO promotes the degradation of EREG mRNA. Taken together, these results suggest that FTO suppresses EREG expression by removing its m6A modification, thereby reducing its mRNA stability.

### 3.7 FTO suppresses glioma progression by inactivating the EREG/PI3K/Akt signaling axis

Having established the FTO-EREG link and recalling our KEGG analysis, we hypothesized that FTO exerts its function through the PI3K/Akt pathway, a known downstream target of EREG (Huang et al., 2019). Western blot analysis revealed that FTO knockdown significantly increased the phosphorylation of PI3K and Akt, whereas FTO overexpression had the opposite effect (Figures 7G,H). Furthermore, the expression of key downstream effectors of the PI3K/Akt pathway, the tumor suppressor p53 and the cell cycle inhibitor p21, was decreased upon FTO knockdown and increased upon FTO overexpression (Figures 7G,H).

**FIGURE 8:**
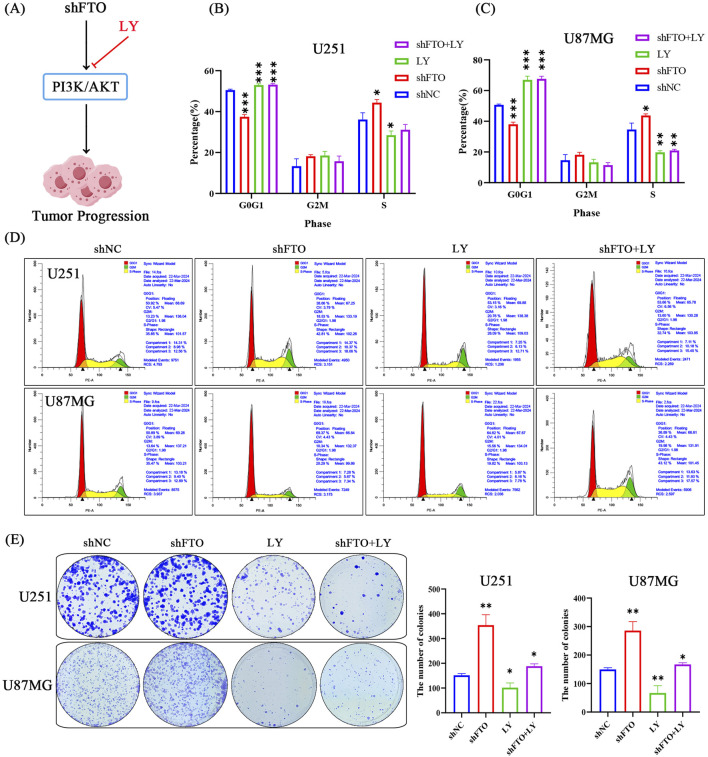
Inhibition of the PI3K/Akt pathway rescues the pro-proliferative phenotype induced by FTO knockdown. **(A)** Schematic diagram illustrating the experimental design to inhibit the PI3K/Akt pathway using LY294002 (LY). **(B–D)** Flow cytometry analysis showing that LY treatment reverses the accelerated G1/S transition caused by FTO knockdown in both U251 **(B)** and U87MG **(C)** cells. Representative plots are shown in **(D)**. **(E)** Representative images and quantification of colony formation assays demonstrating that LY treatment significantly abrogates the enhanced clonogenic growth induced by FTO knockdown. Data are shown as mean ± SD from three independent experiments. *p < 0.05, **p < 0.01, ***p < 0.001.

To confirm the causal role of this pathway, we treated FTO-knockdown cells with the PI3K inhibitor LY294002 (LY) (Figure 8A). Treatment with LY successfully reversed the accelerated cell proliferation and G1-S transition induced by FTO knockdown, as demonstrated by colony formation and cell cycle analyses (Figures 8B–E). These rescue experiments confirm that FTO suppresses glioma cell proliferation and cell cycle progression by inhibiting the PI3K/Akt signaling axis.

## 4 Discussion

The aggressive proliferation of glioma cells is a major obstacle to effective treatment, making the identification of key regulatory molecules a priority for developing new therapies. In this study, we definitively establish the m6A demethylase FTO as a critical tumor suppressor in glioma. Through a comprehensive approach combining large-scale clinical data analysis, *in vitro* functional assays, and an *in vivo* xenograft model, we demonstrate that a FTO-EREG-PI3K/Akt signaling axis plays a pivotal role in controlling glioma proliferation.

Our initial analysis of over 1,000 glioma patients from the TCGA and CGGA databases, corroborated by our own clinical samples, consistently showed that FTO expression is significantly downregulated in high-grade gliomas and is strongly associated with poor overall survival. This finding aligns with previous reports that also link low FTO levels to glioma malignancy (Chai et al., 2019; Tao et al., 2020; Zhang et al., 2022), solidifying its potential as a prognostic biomarker. The integration of FTO expression into multivariable prognostic models, alongside established markers like IDH status and MGMT promoter methylation, could potentially enhance risk stratification and guide personalized treatment decisions for glioma patients (Brat et al., 2015). However, we recognize that our survival analysis relies on univariate Kaplan-Meier curves. Future studies incorporating multivariate Cox regression analysis are needed to confirm FTO as an independent prognostic factor after adjusting for key clinical confounders, such as age and IDH mutation status (Parsons et al., 2008). While some studies have focused on inhibiting FTO in certain cancers where it functions as an oncogene, such as in acute myeloid leukemia (Huang et al., 2019; Niu et al., 2019), our data clearly position FTO as a protective factor in the context of glioma, where its loss contributes to disease progression. This highlights the critical, context-dependent function of m6A regulators in tumorigenesis, cautioning against a one-size-fits-all therapeutic approach targeting these enzymes across different malignancies.

The tumor-suppressive function of FTO was mechanistically dissected in our study. We demonstrated that FTO-mediated suppression of proliferation is intrinsically linked to its ability to induce G1 phase cell cycle arrest. This is a hallmark of many tumor suppressors that act as gatekeepers against uncontrolled cell division. Our investigation into the underlying mechanism led us to identify EREG as a novel and critical downstream target of FTO in glioma. EREG is a known ligand for the epidermal growth factor receptor (EGFR), and its overexpression has been implicated in promoting proliferation in various cancers (Chen et al., 2022; Gonzalez-Conchas et al., 2018). The EGFR pathway is notoriously dysregulated in a significant subset of GBM, often through gene amplification or mutation (van den Bent et al., 2015; Kuan et al., 2001; Nakada et al., 2011), making the FTO-EREG link a potentially critical upstream regulatory event that fine-tunes EGFR signaling strength (Schmidt et al., 2003; Sunaga and Kaira, 2015). Our data suggest a novel regulatory mechanism where FTO, through its m6A demethylase activity, destabilizes EREG mRNA, leading to its downregulation. The loss of FTO in aggressive glioma would therefore lead to the stabilization and accumulation of EREG mRNA, fueling a proliferative signaling cascade.

This cascade culminates in the activation of the PI3K/Akt pathway, a central hub for cell growth, survival, and metabolism that is frequently hyperactivated in glioma (Barzegar Behrooz et al., 2022). Our results show that loss of FTO enhances PI3K/Akt phosphorylation, which in turn leads to the downregulation of the tumor suppressor p53 and the cyclin-dependent kinase inhibitor p21. The p53/p21 axis is a canonical downstream effector that enforces cell cycle checkpoints (Engeland, 2022). This finding is particularly relevant as the interplay between the PI3K/Akt/mTOR and p53 pathways is a critical determinant of cell fate, and their co-dysregulation is a common feature of glioma progression and therapy resistance (Barzegar Behrooz et al., 2022; Kuduvalli et al., 2023; Mao et al., 2012). Its suppression by the FTO-knockdown-induced PI3K/Akt activation explains the accelerated G1/S transition we observed. The successful rescue of the proliferative phenotype by the PI3K inhibitor LY294002 provides strong evidence for the crucial role of this pathway downstream of FTO.

**FIGURE 9:**
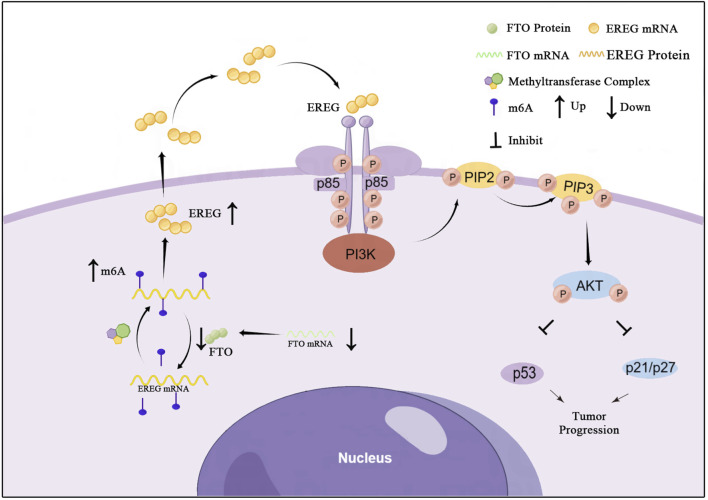
A proposed model for the tumor-suppressive role of FTO in glioma. In normal glial cells or low-grade glioma, FTO is expressed and functions to demethylate EREG mRNA, leading to its degradation. This limits EREG expression, keeping the PI3K/Akt pathway in check and allowing p53/p21 to enforce cell cycle control, thereby suppressing proliferation. In high-grade glioma, FTO is downregulated. This leads to hypermethylation (↑m6A) and stabilization of EREG mRNA, resulting in EREG protein upregulation. The excess EREG activates the PI3K/Akt signaling cascade, which suppresses p53 and p21, leading to uncontrolled cell cycle progression and tumor growth.

In summary, our study delineates a clear tumor-suppressive pathway in glioma: FTO demethylates and destabilizes EREG mRNA, which limits EREG-mediated activation of the PI3K/Akt pathway. This maintains the expression of p53 and p21, thereby restraining cell proliferation and cell cycle progression (Figure 9). The downregulation of FTO in high-grade glioma disrupts this regulatory brake, leading to uncontrolled tumor growth.

Despite the strengths of our study, we acknowledge several limitations that temper our conclusions and highlight avenues for future investigation. First, while our inhibitor and mRNA stability assays provide strong indirect evidence for m6A-dependent regulation, the absence of a direct binding assay, such as m6A-RNA immunoprecipitation (MeRIP-qPCR), means we have not definitively proven that FTO directly demethylates EREG mRNA. This experiment remains the gold standard to confirm a direct physical interaction. Similarly, a rescue experiment employing an shRNA-resistant FTO construct would have further solidified the specificity of our knockdown findings by ruling out potential off-target effects. Second, our *in vivo* experiments were conducted using a subcutaneous model, which, while valuable for assessing tumor growth, does not fully recapitulate the unique microenvironment of an orthotopic intracranial tumor. The complex interplay with the blood-brain barrier and brain-resident cells is a critical aspect of glioma biology that our model does not address. Therefore, validating our findings in clinically relevant models, such as patient-derived orthotopic xenografts (PDOXs) or glioma organoids, is a necessary next step (Klein et al., 2020; Oudin et al., 2021; Xu and Li, 2021). Third, while our findings highlight the therapeutic potential of restoring FTO function, developing FTO agonists remains a significant pharmacological challenge. A more immediately translatable strategy could involve the targeted inhibition of downstream effectors. For instance, therapies like EREG monoclonal antibodies or selective PI3K/Akt inhibitors, many of which are in clinical development, could be repurposed for glioma subtypes characterized by low FTO expression (Li et al., 2016; Wick et al., 2011; Roth et al., 2014). Future research should focus on validating the FTO-EREG axis in orthotopic models and exploring these therapeutic strategies.

## Data Availability

The datasets presented in this study can be found in online repositories. The names of the repository/repositories and accession number(s) can be found in the article/Supplementary Material.

## Abbreviations

m6A, N6-methyladenosine; FTO, Fat mass and obesity-associated protein; EREG, Epiregulin; PI3K, Phosphoinositide 3-kinase; Akt, Protein kinase B; GBM, Glioblastoma multiforme; TCGA, The Cancer Genome Atlas; CGGA, Chinese Glioma Genome Atlas; GO, Gene Ontology; KEGG, Kyoto Encyclopedia of Genes and Genomes; DEGs, Differentially Expressed Genes; qRT-PCR, Quantitative real-time polymerase chain reaction; IHC, Immunohistochemistry; EdU, 5-Ethynyl-2′-deoxyuridine; shRNA, Short hairpin RNA; oe, Overexpression (e.g., oeFTO); LY, LY294002 (PI3K inhibitor); IOD, Integrated Optical Density.
