# Supplementary material for: The m6A demethylase FTO suppresses glioma proliferation by regulating the EREG/PI3K/Akt signaling pathway
Source: Front Cell Dev Biol. 2025 Sep 3;13:1667990. doi: 10.3389/fcell.2025.1667990 (PMC12441170; doi:10.3389/fcell.2025.1667990)

**glioma tissue samples**

FTO

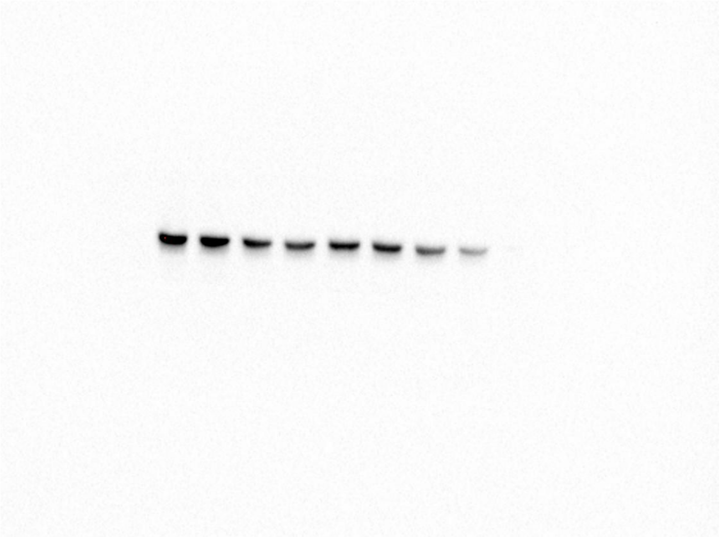

GAPDH

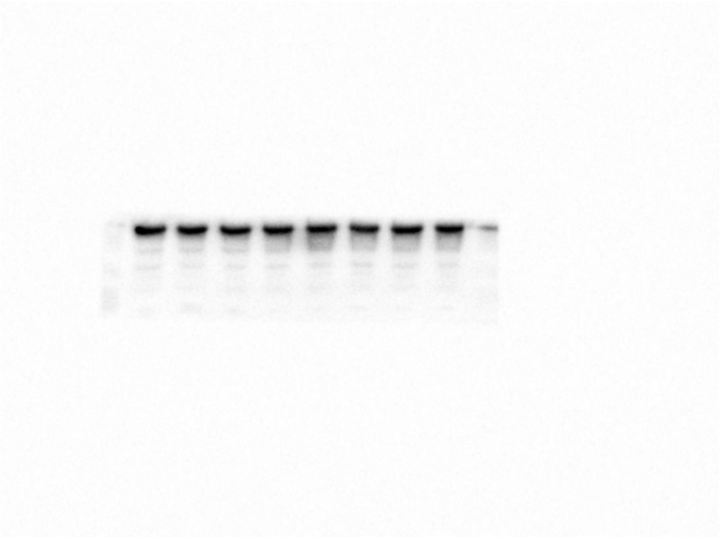

FTO

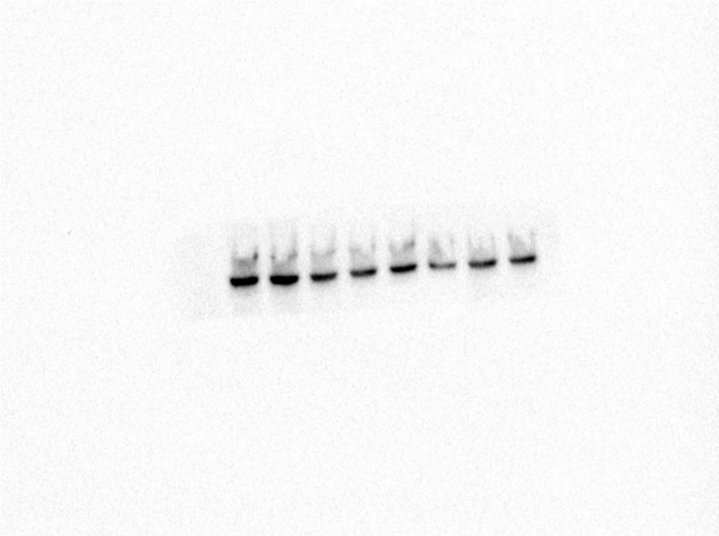

GAPDH

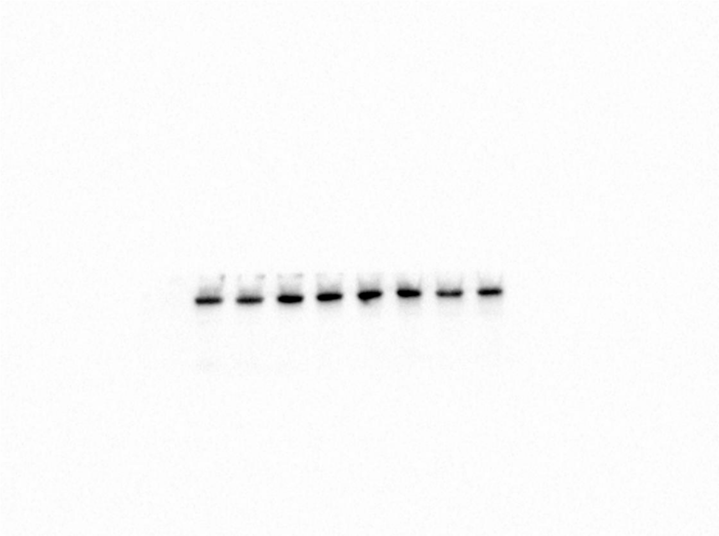

FTO

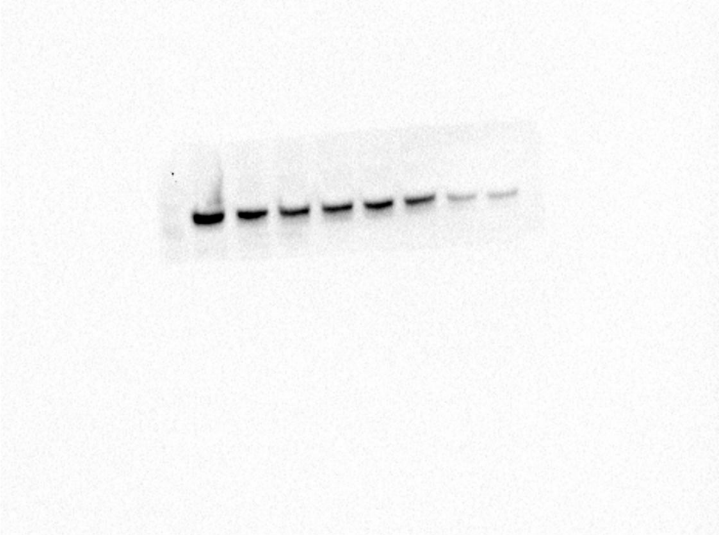

GAPDH

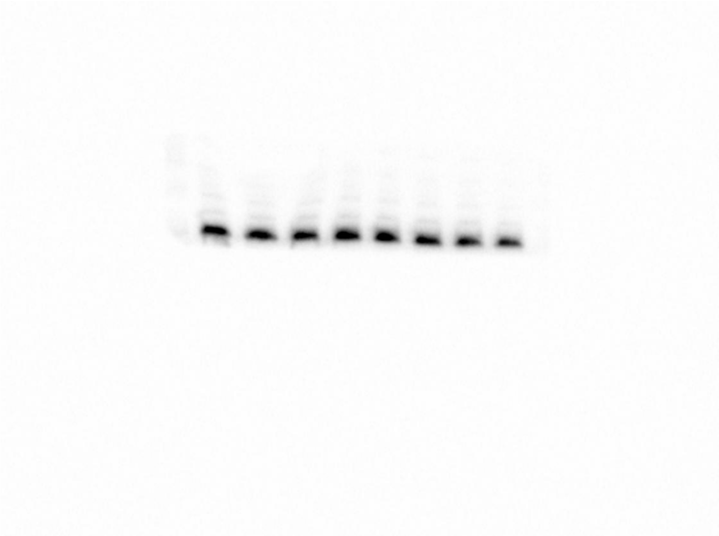

**glioma cell lines**

FTO

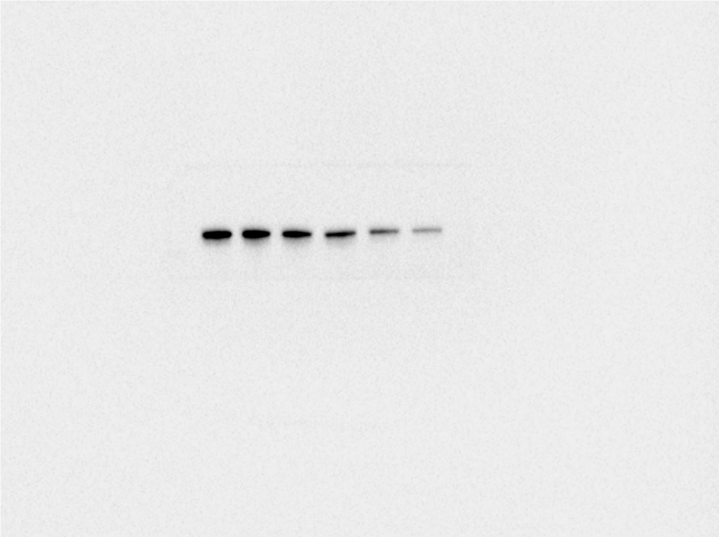

GAPDH

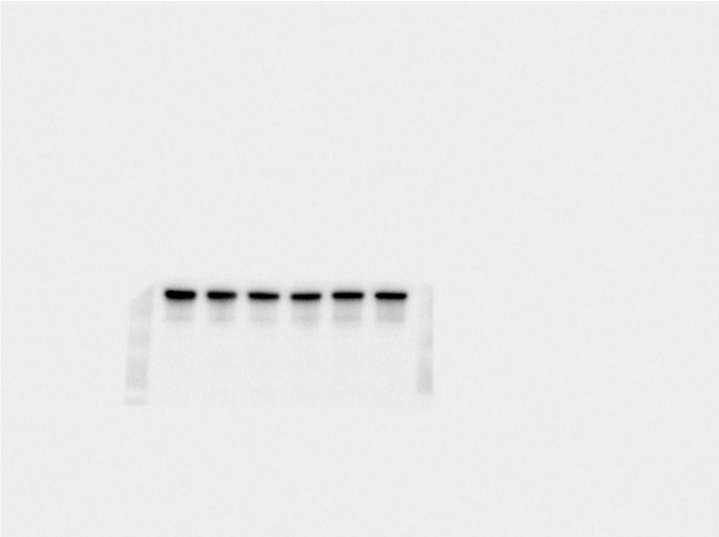

FTO

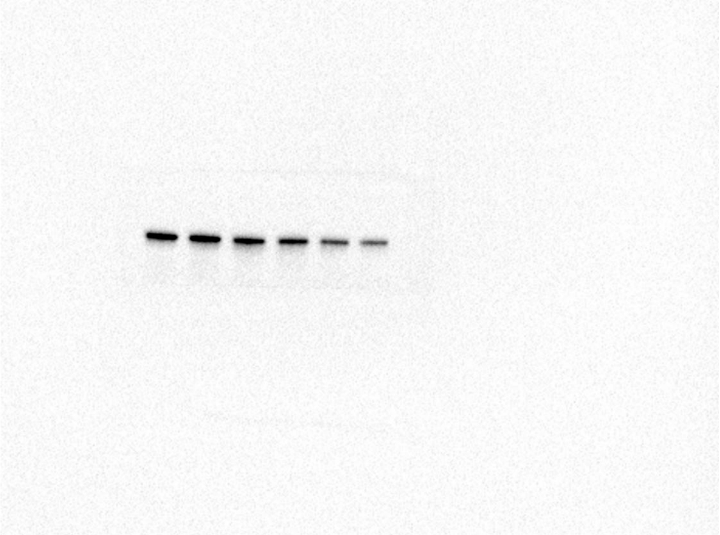

GAPDH

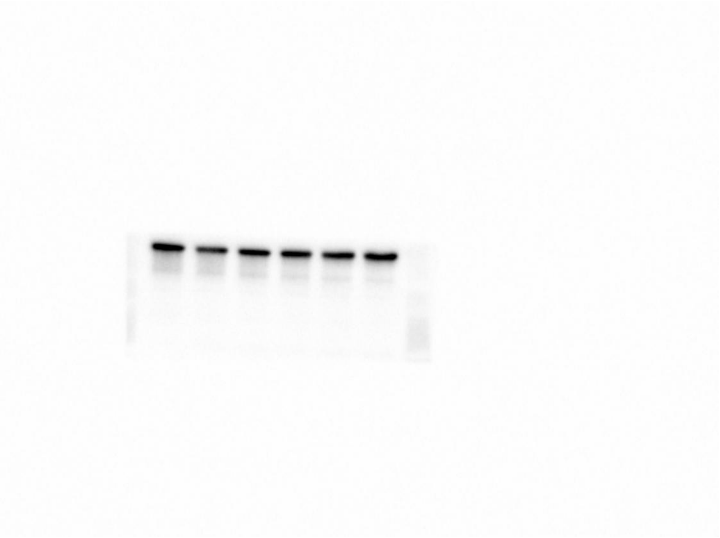

FTO

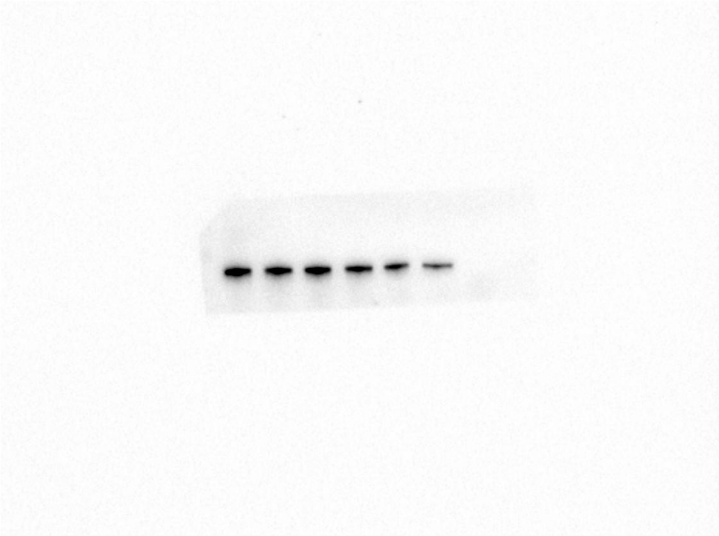

GAPDH

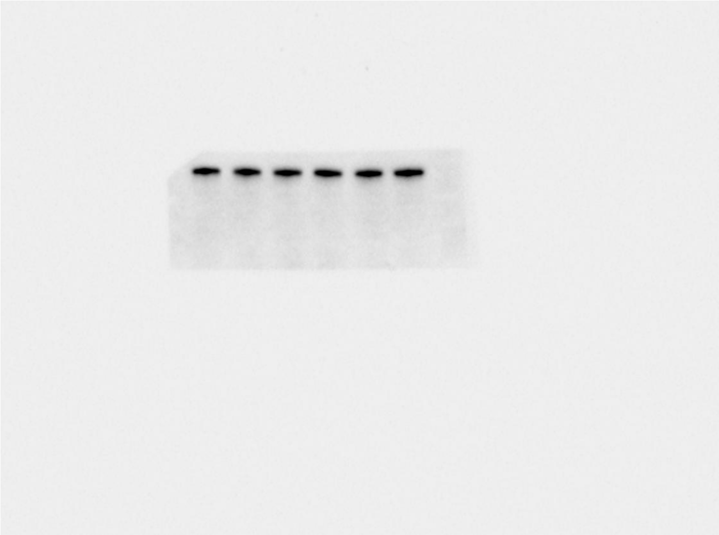

U87MG FTO

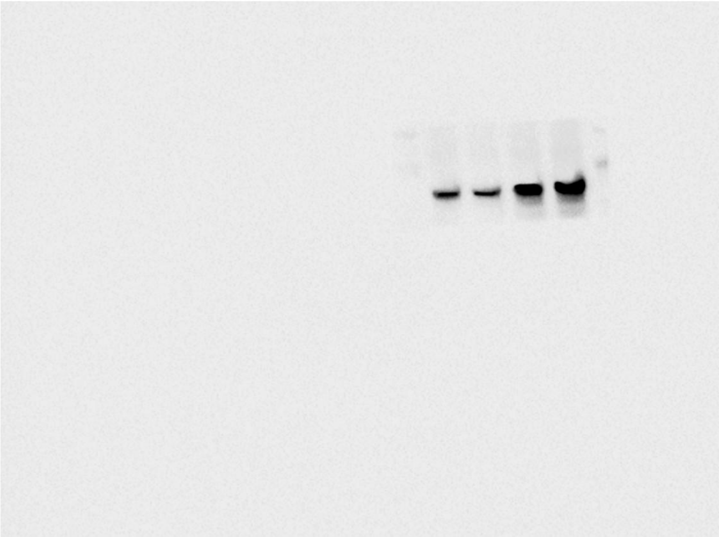

U87MG GAPDH

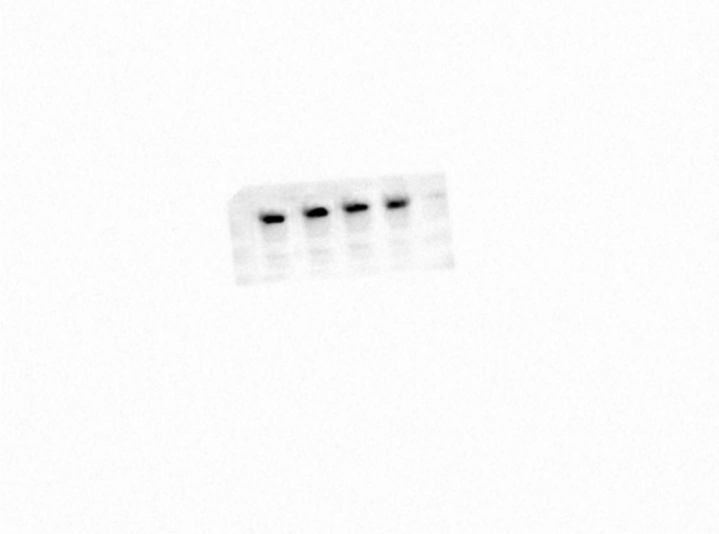

U87MG GAPDH

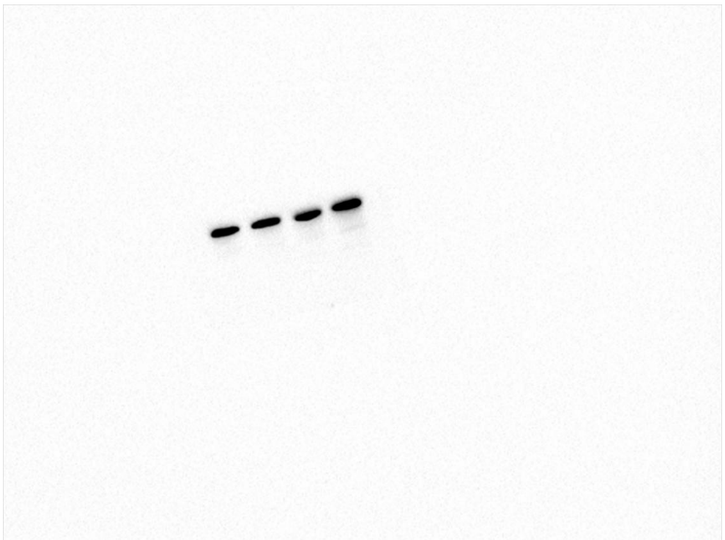

U87MG FTO

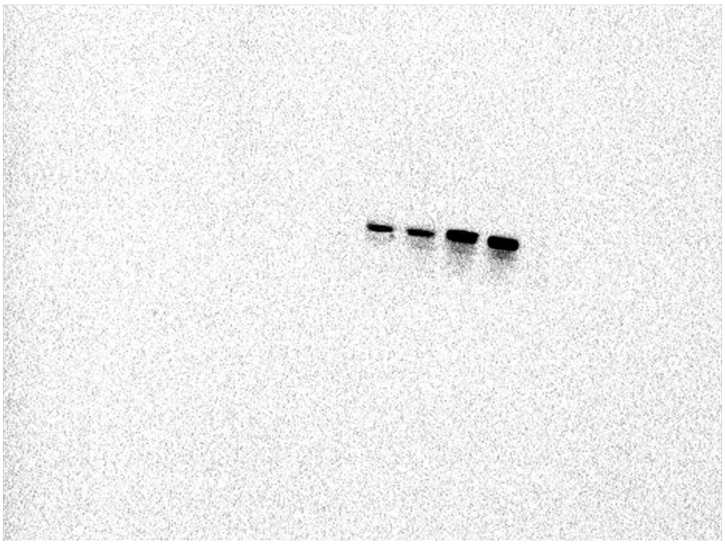

U87MG GAPDH

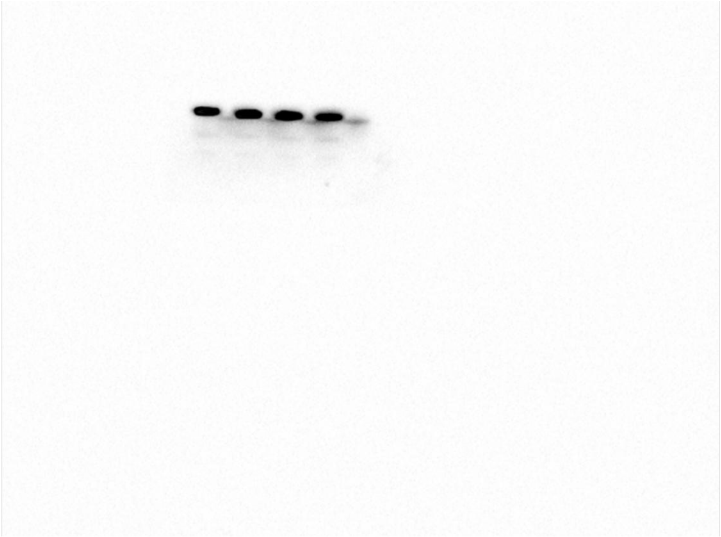

U87MG FTO

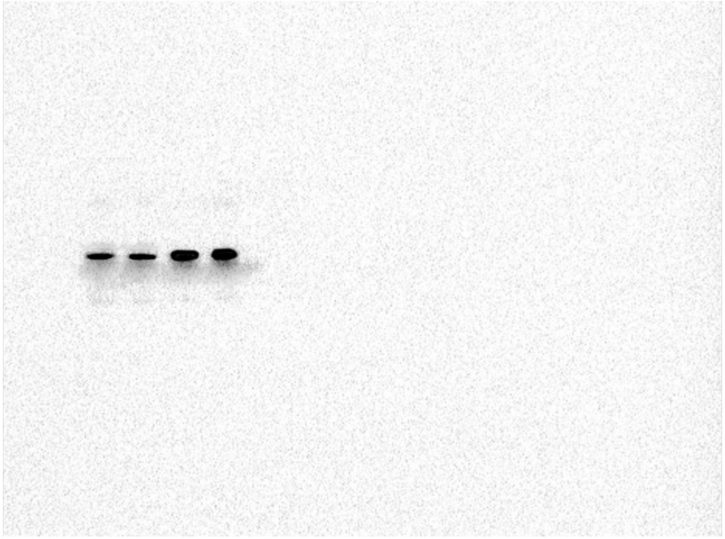

U251 FTO

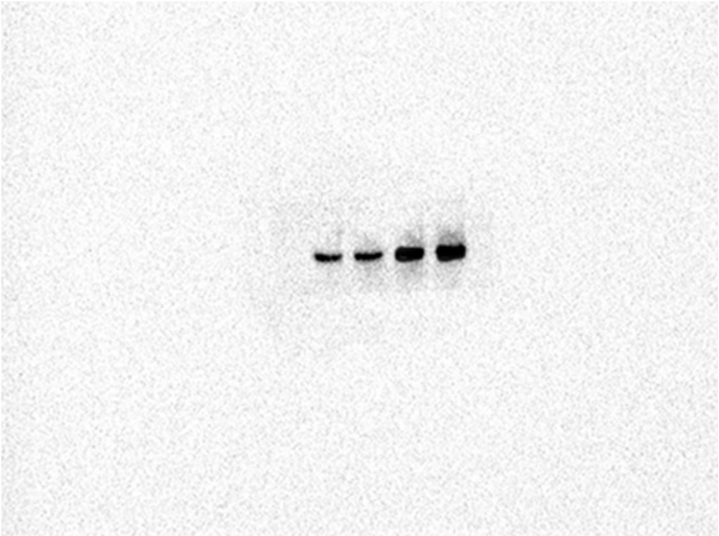

U251 GAPDH

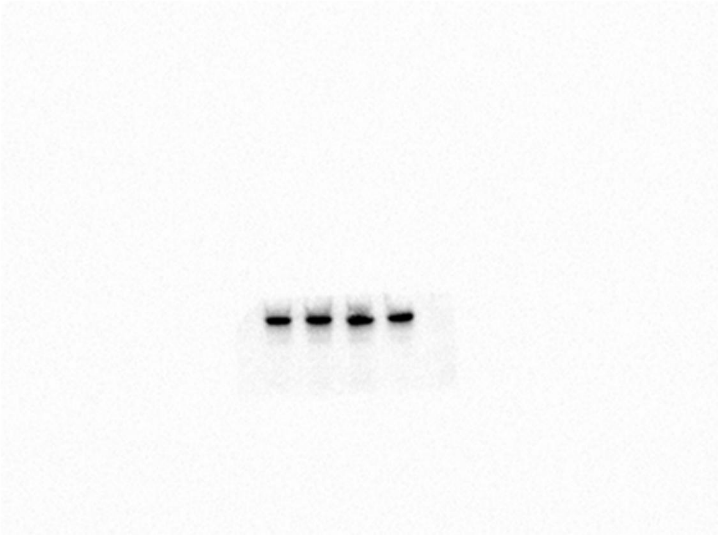

U251 FTO

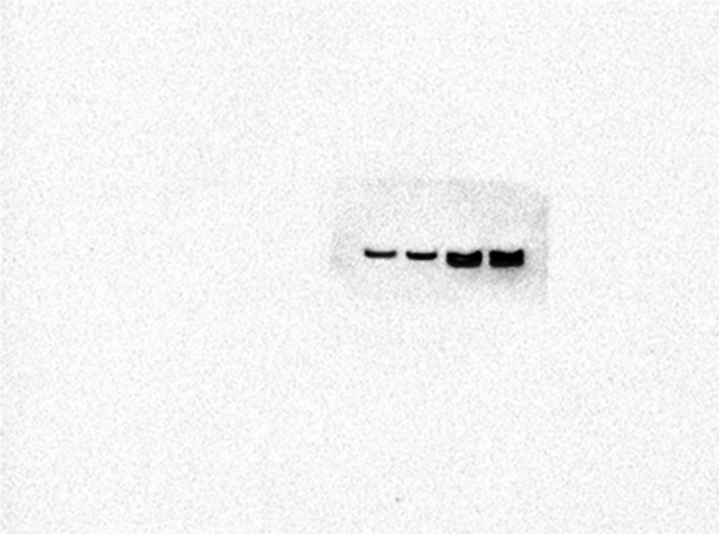

U251 GAPDH

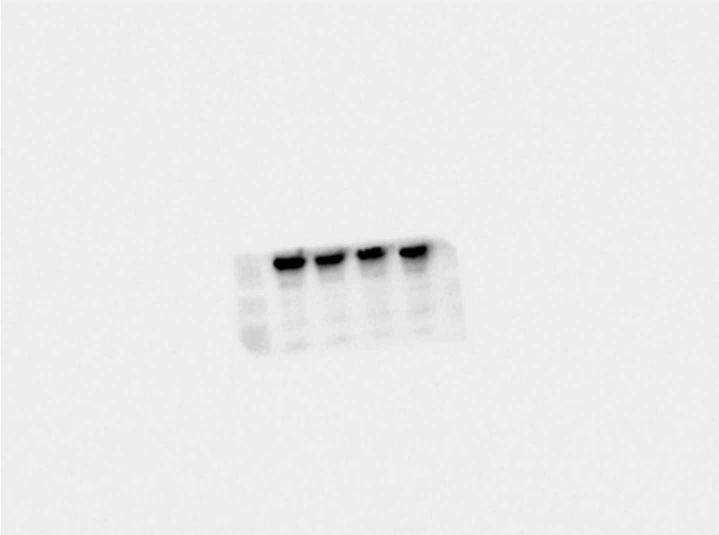

U251 FTO

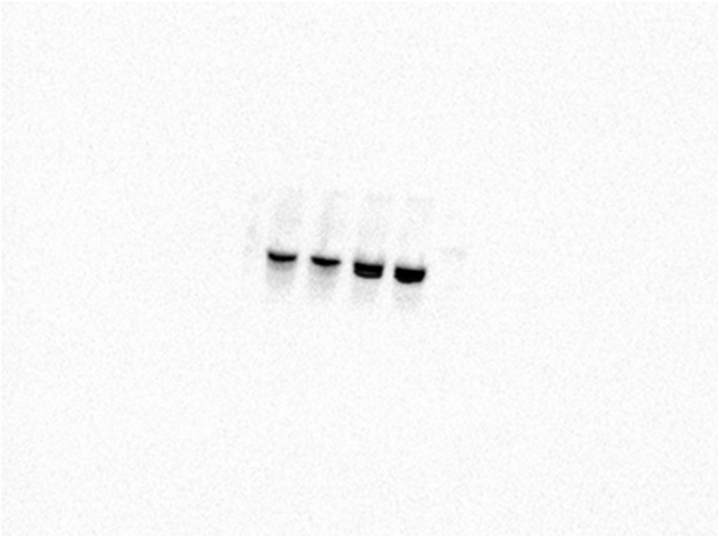

U251 GAPDH

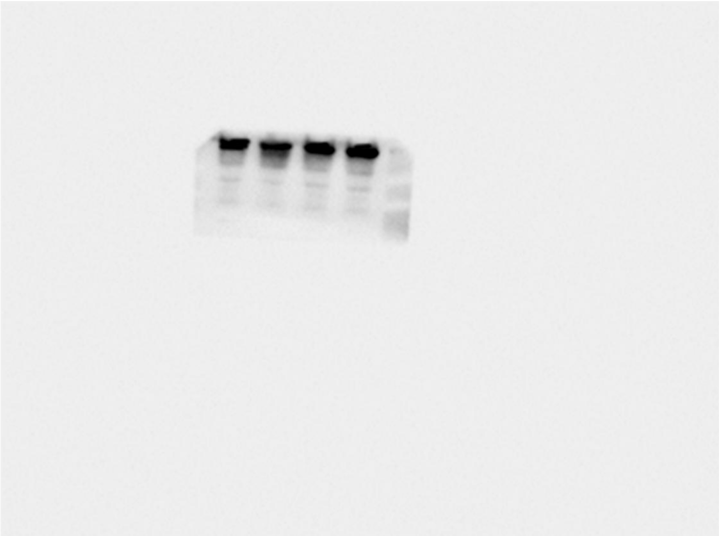

FTO

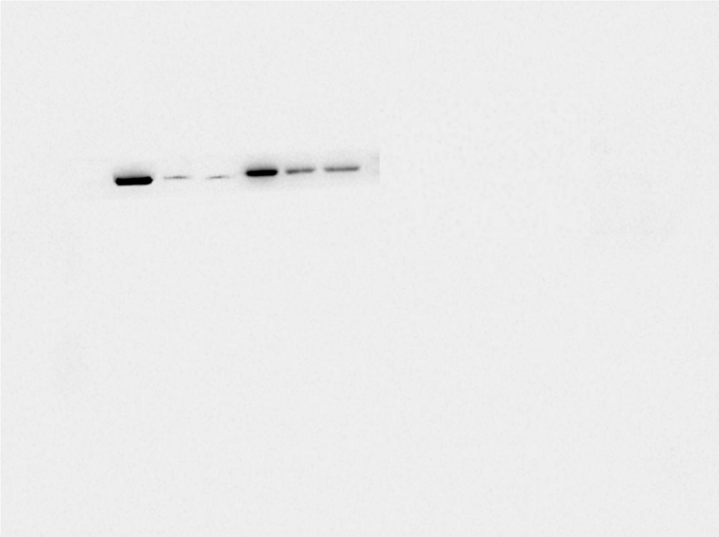

GAPDH

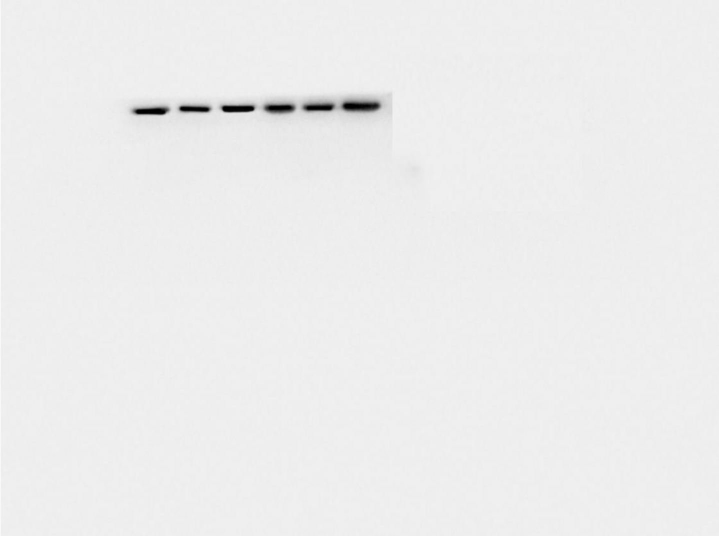

FTO

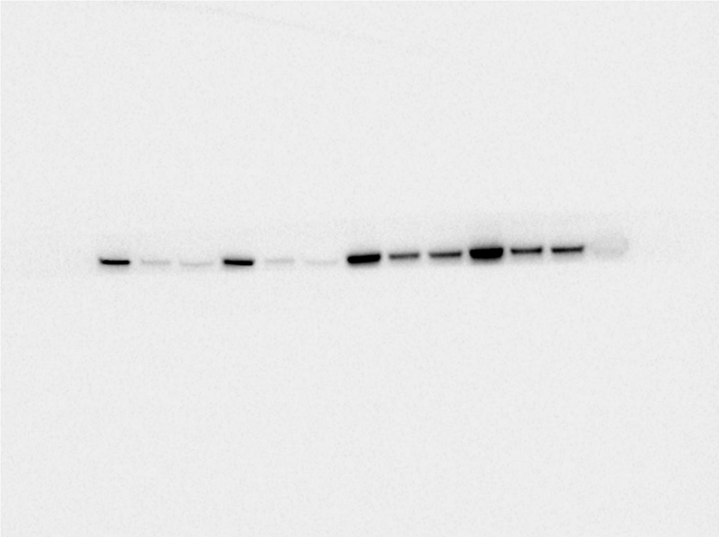

GAPDH

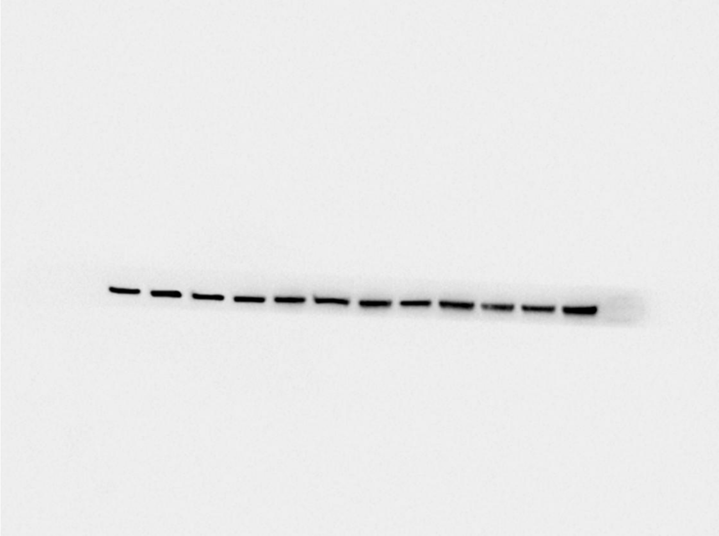

FB23-2

EREG

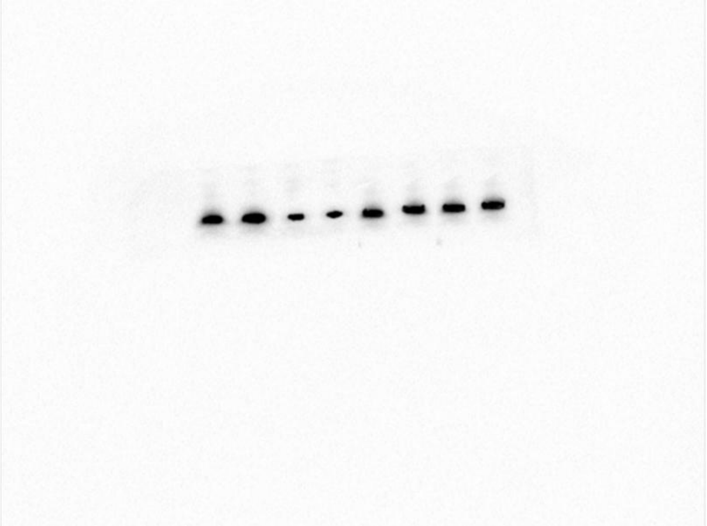

FTO

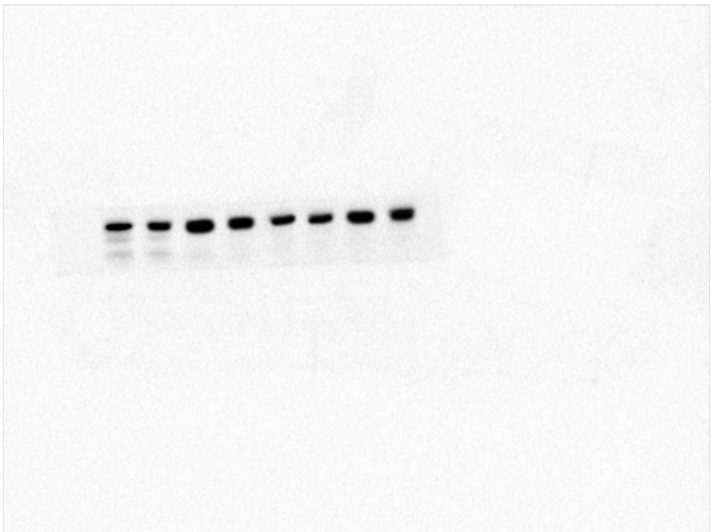

GAPDH

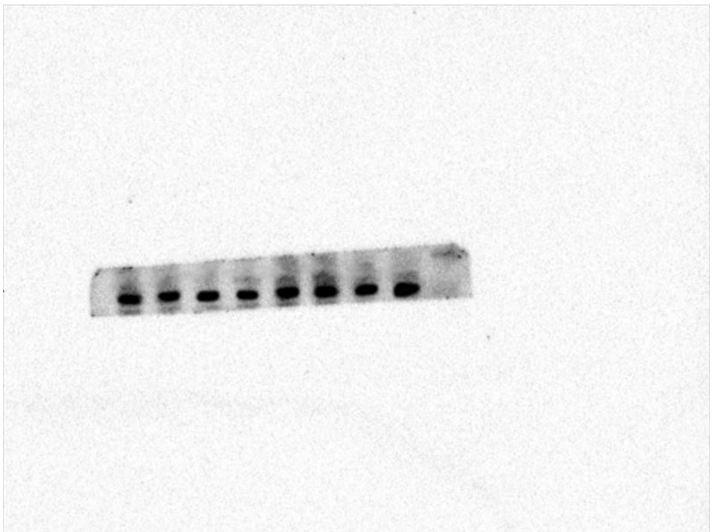

EREG

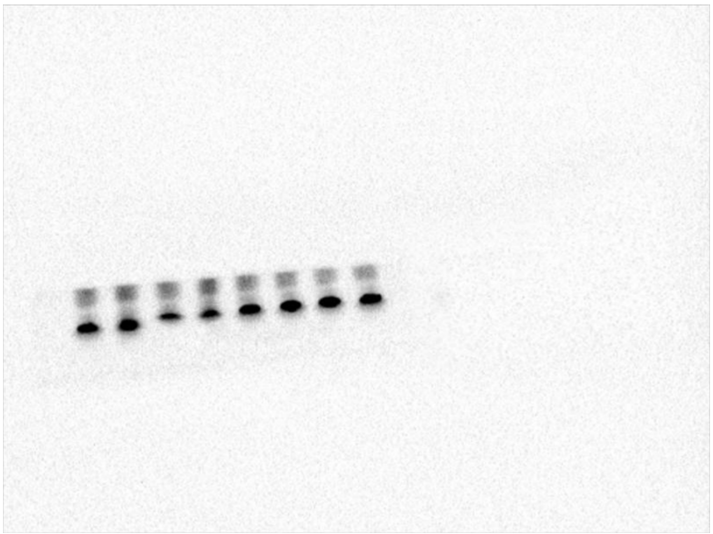

FTO

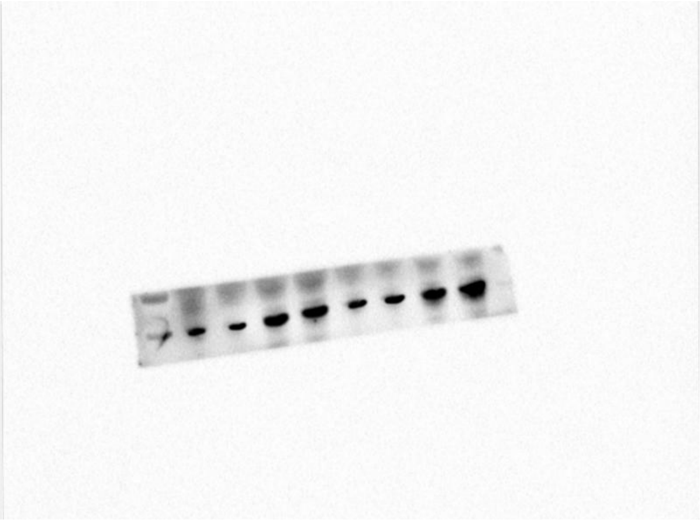

GAPDH

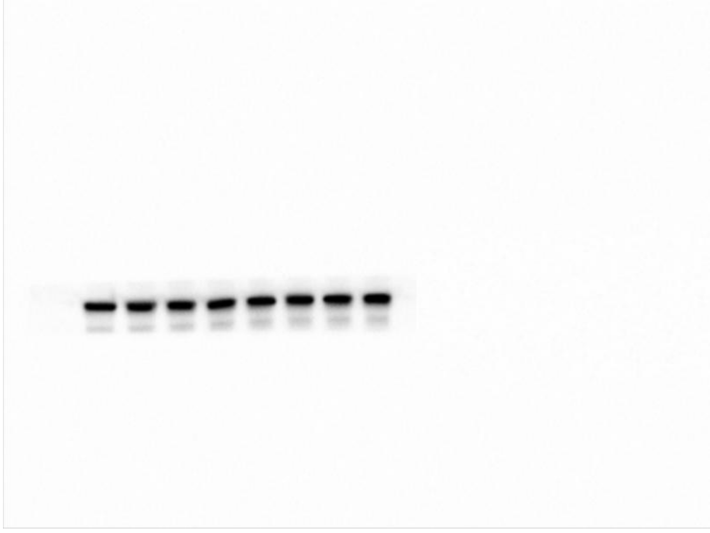

EREG

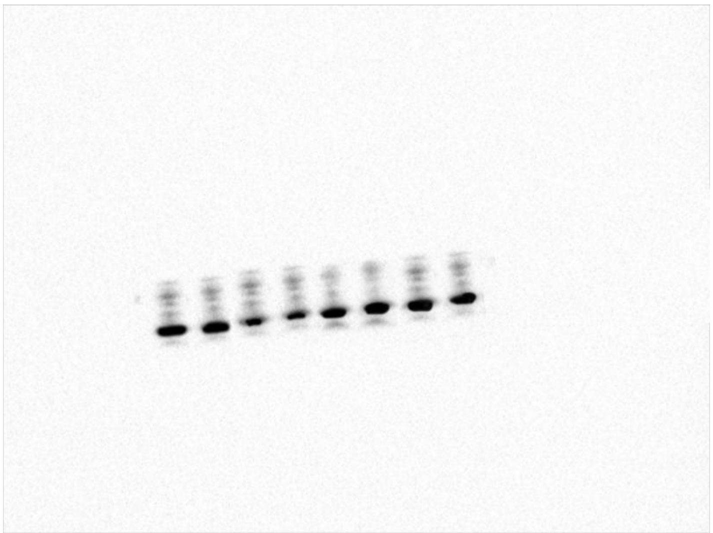

FTO

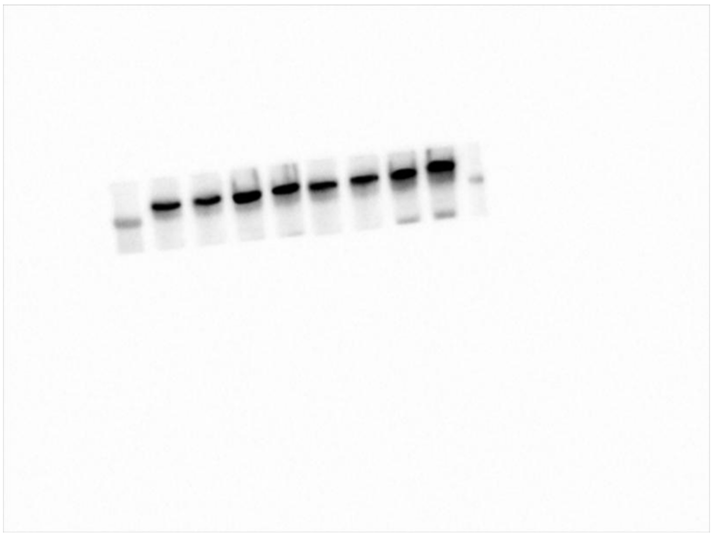

GAPDH

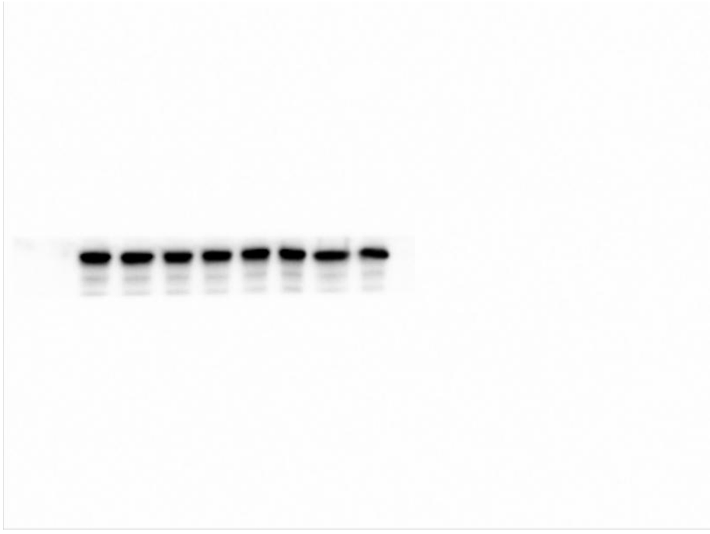

EREG

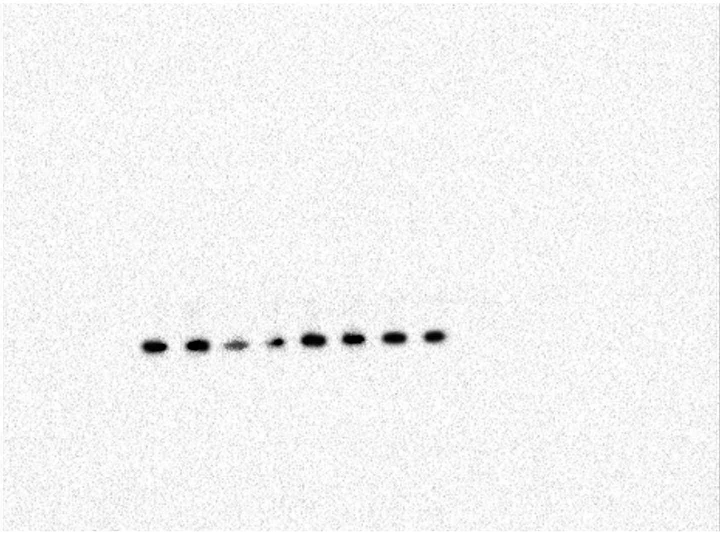

FTO

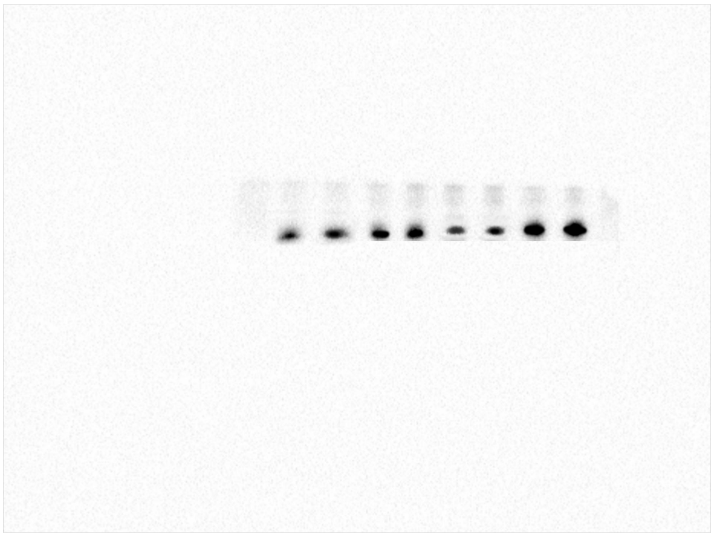

GAPDH

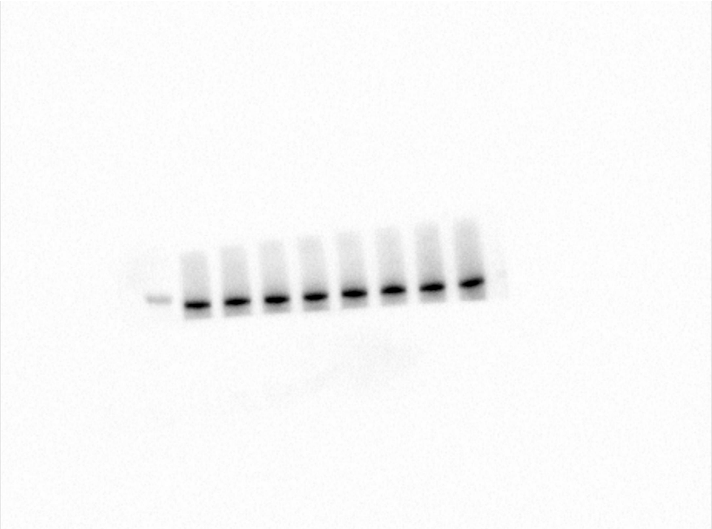

the PI3K/Akt Signaling Pathway

P21

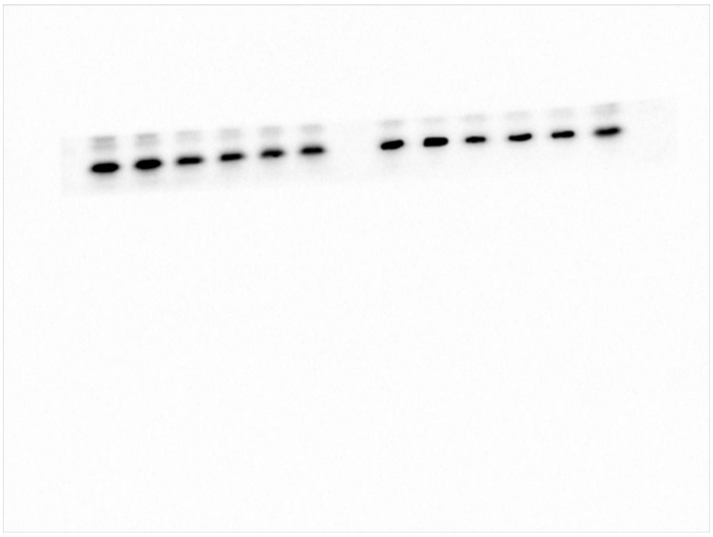

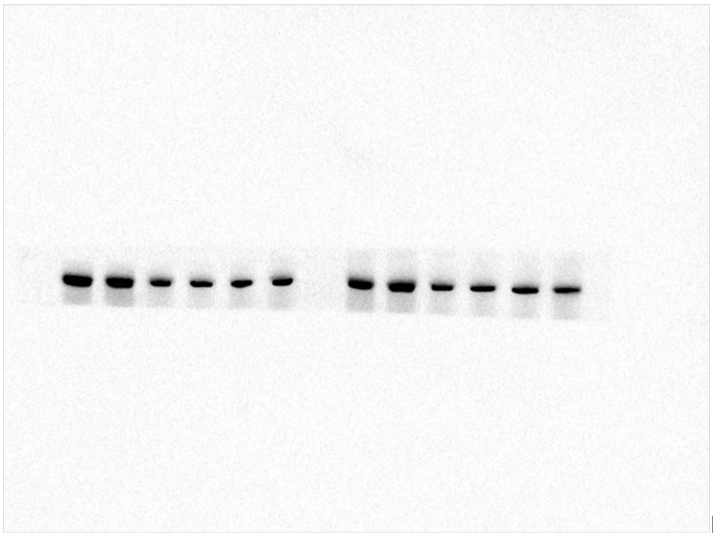

P53

PI3K

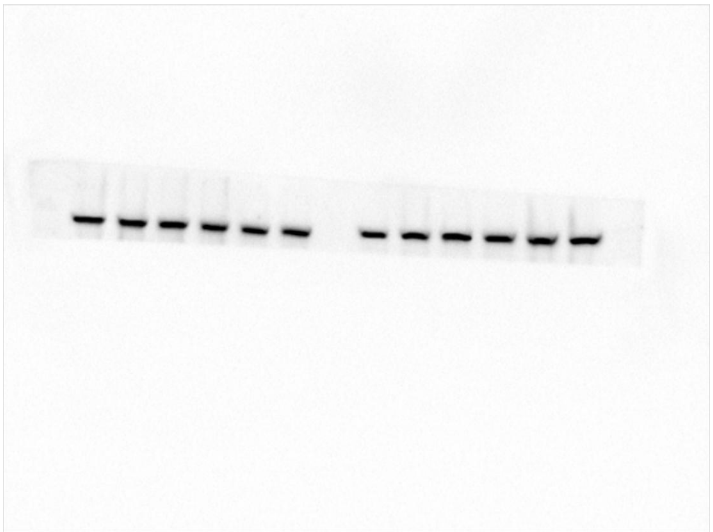

GAPDH

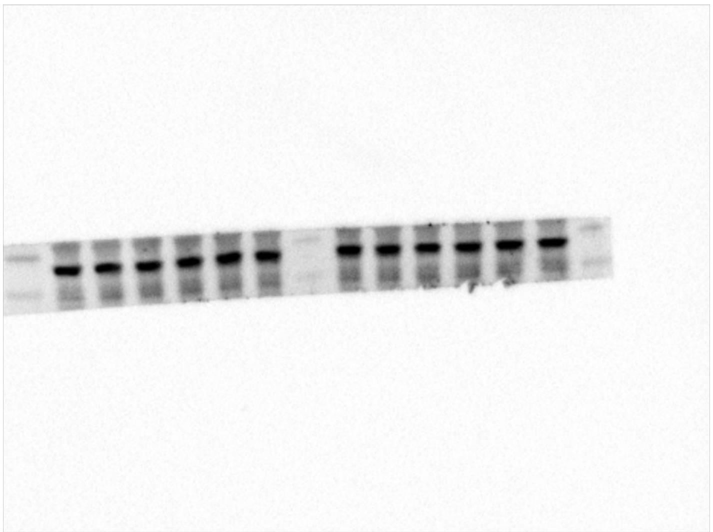

P21

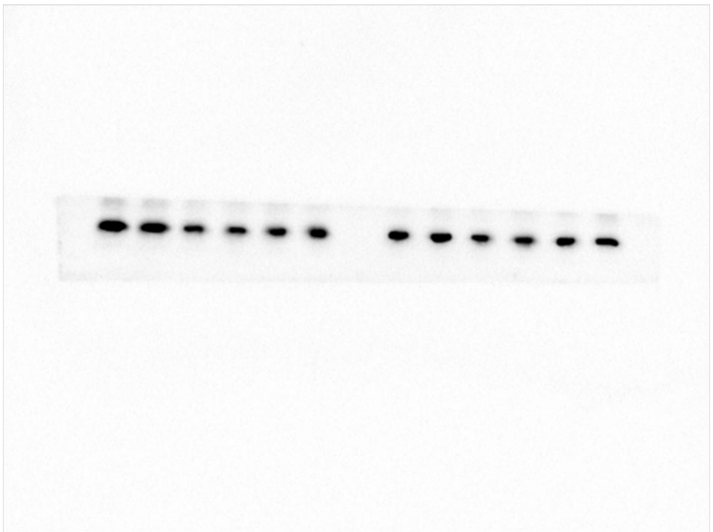

P53

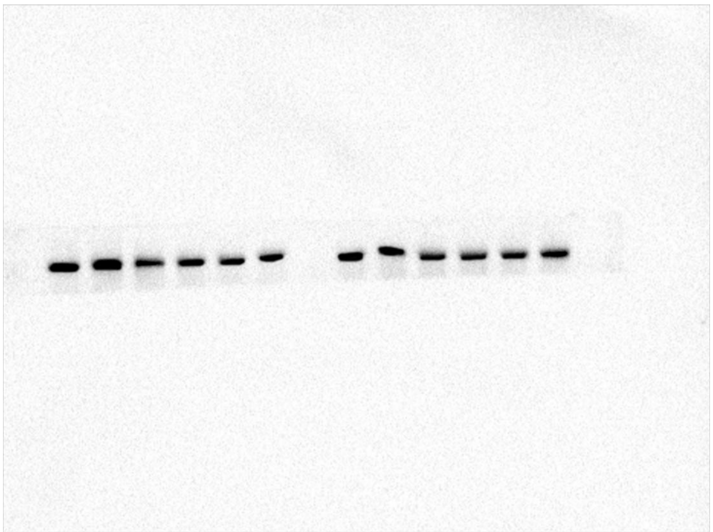

PI3K

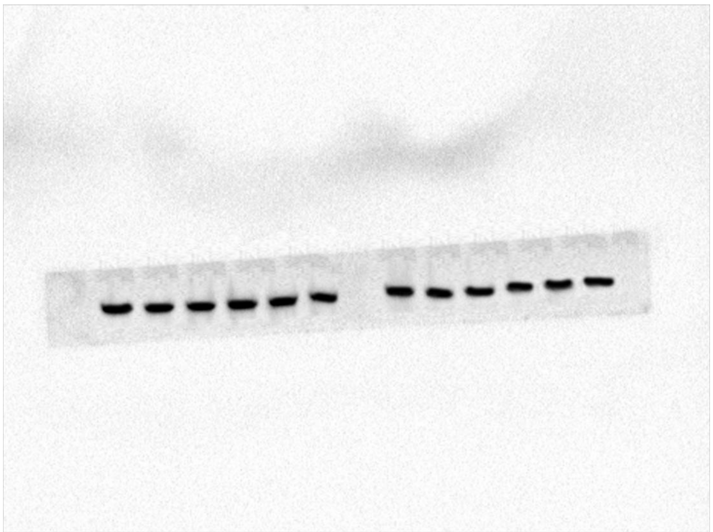

GAPDH

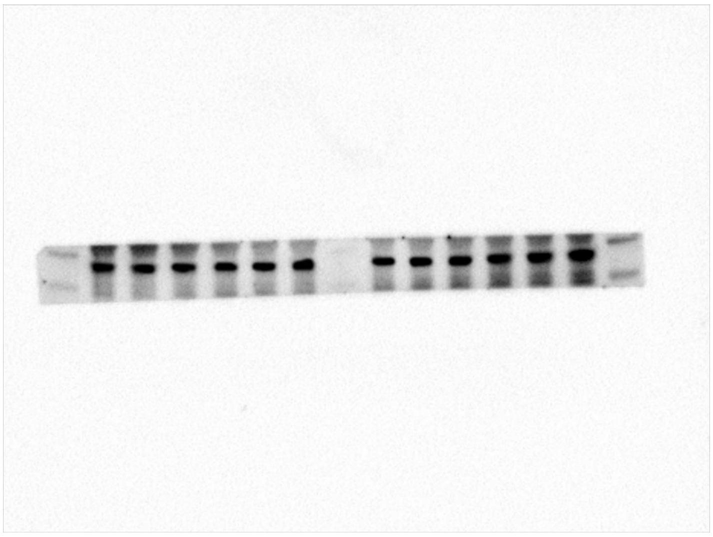

P-PI3K

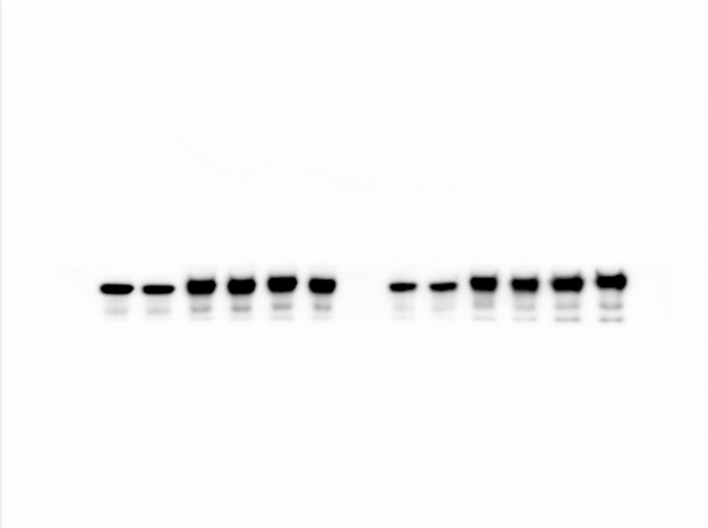

AKT

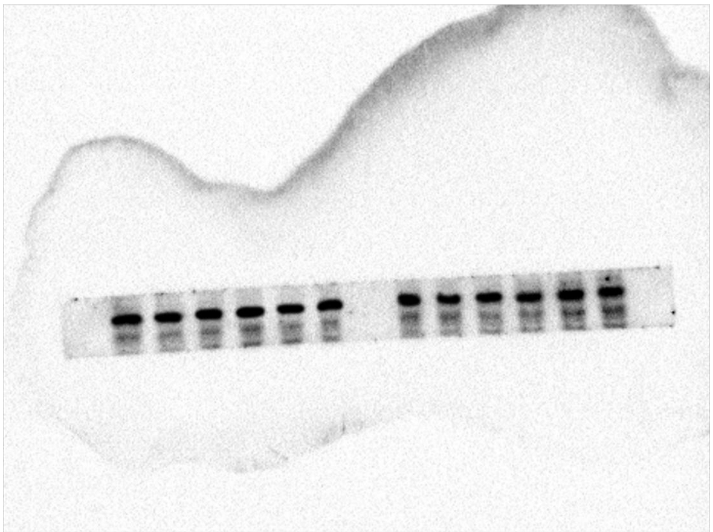

GAPDH

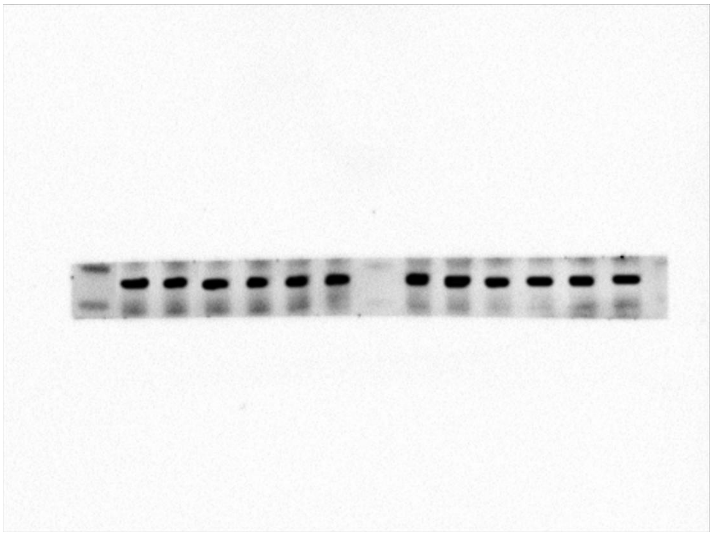

P-PI3K

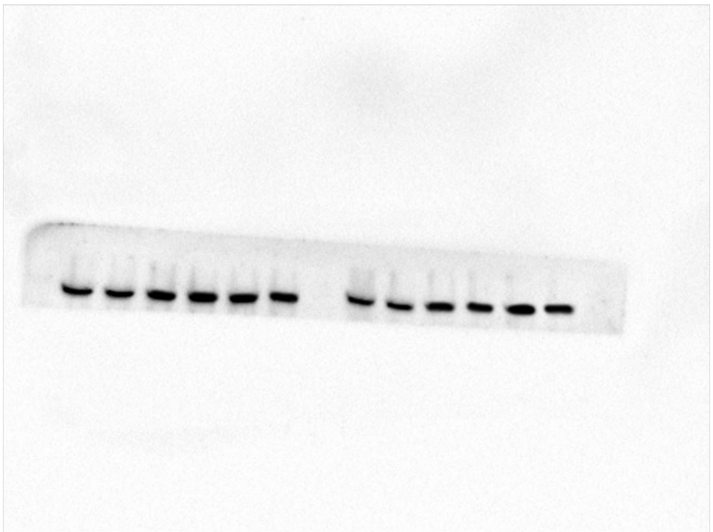

AKT

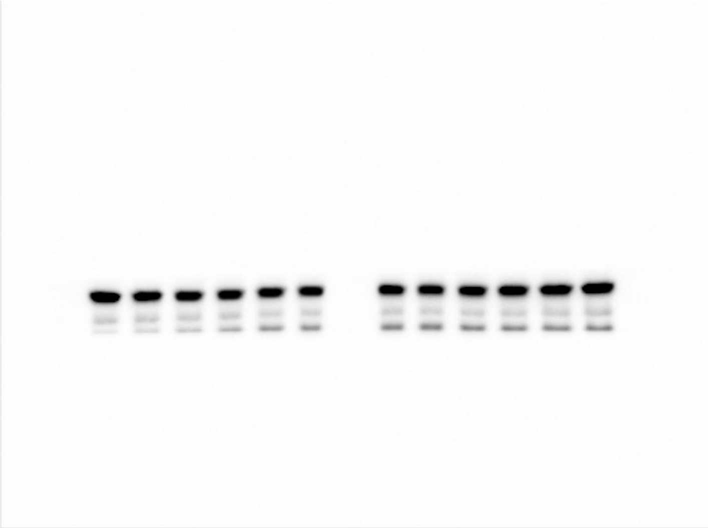

GAPDH

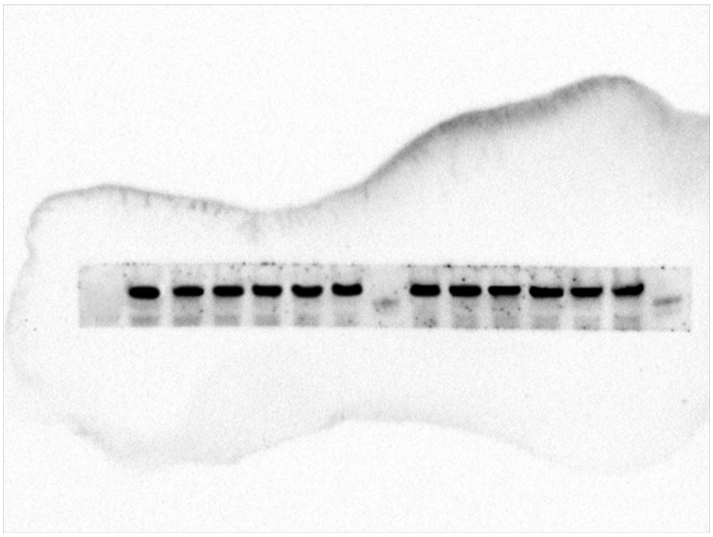

P-AKT

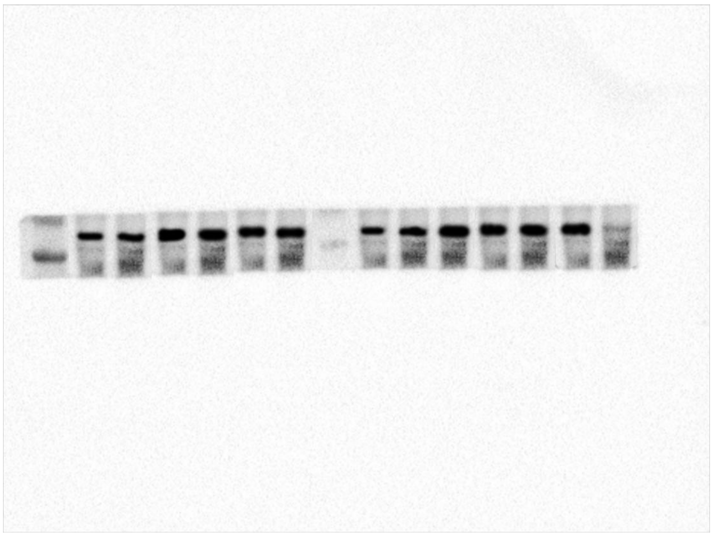

GAPDH

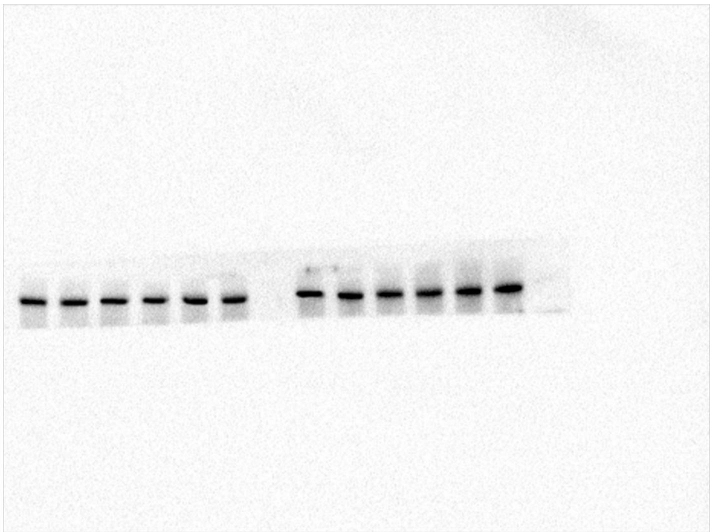

P-AKT

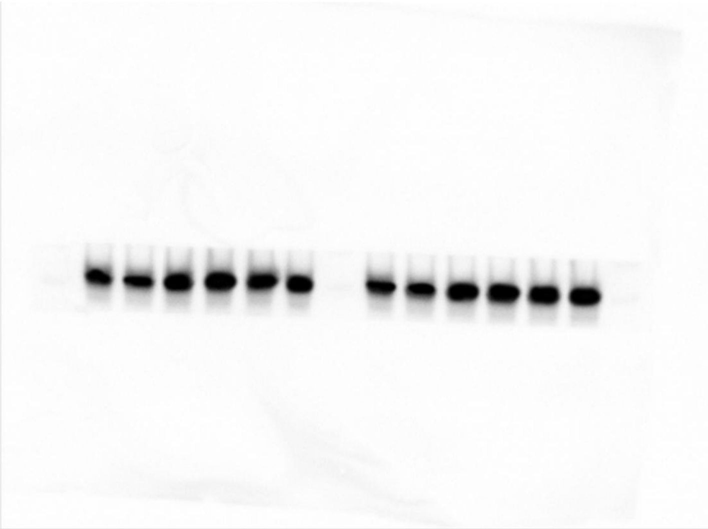

GAPDH

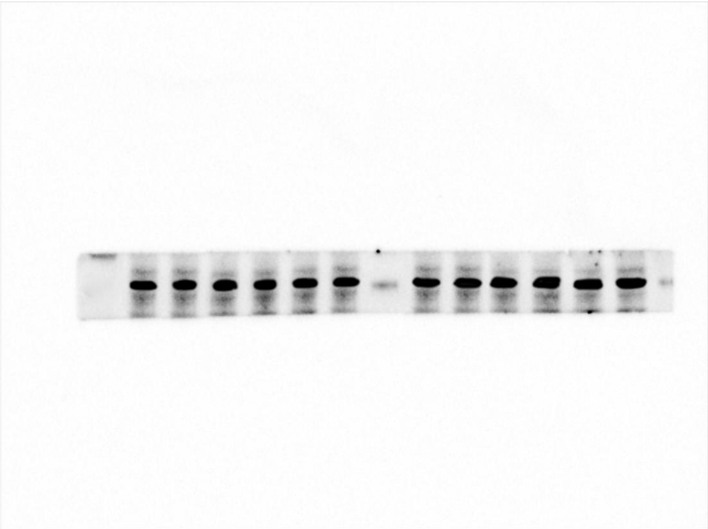

P21

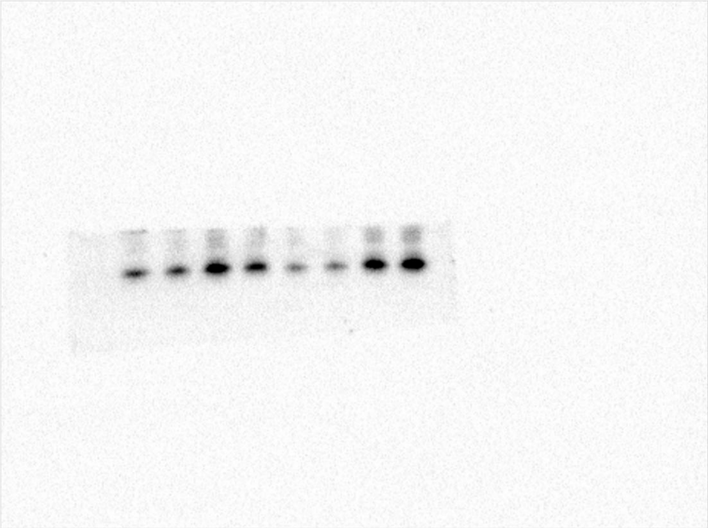

P53

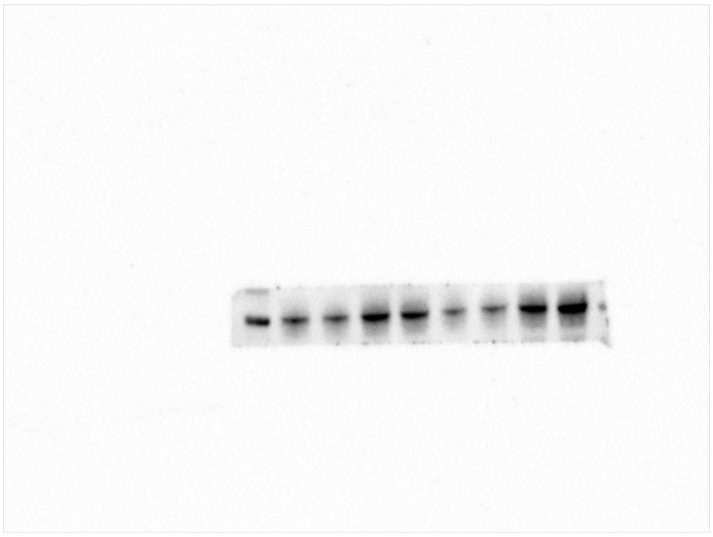

PI3K

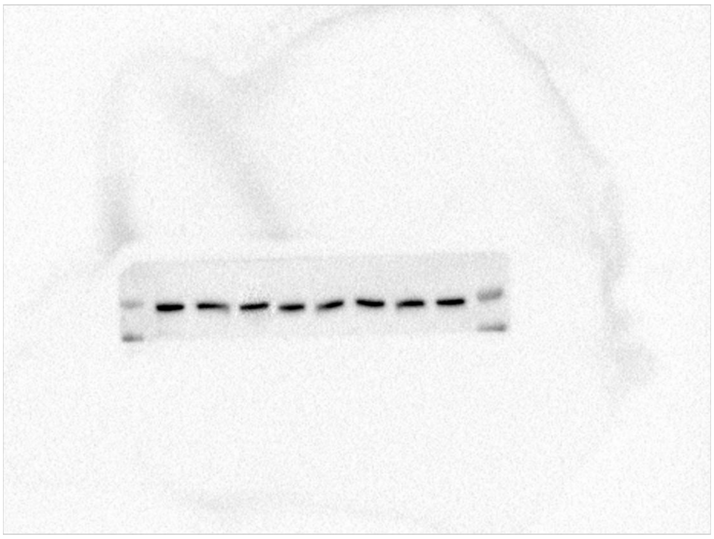

GAPDH

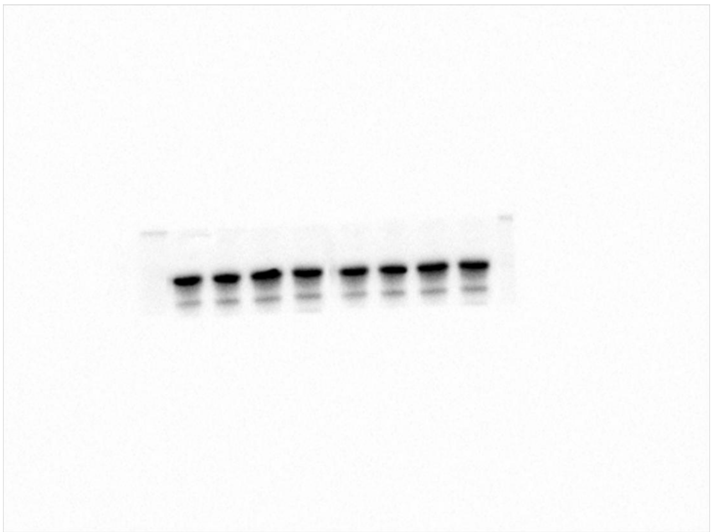

P21

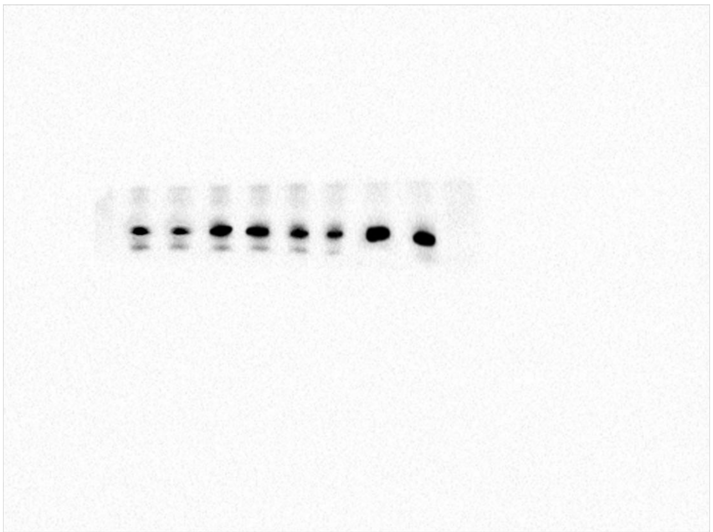

P53

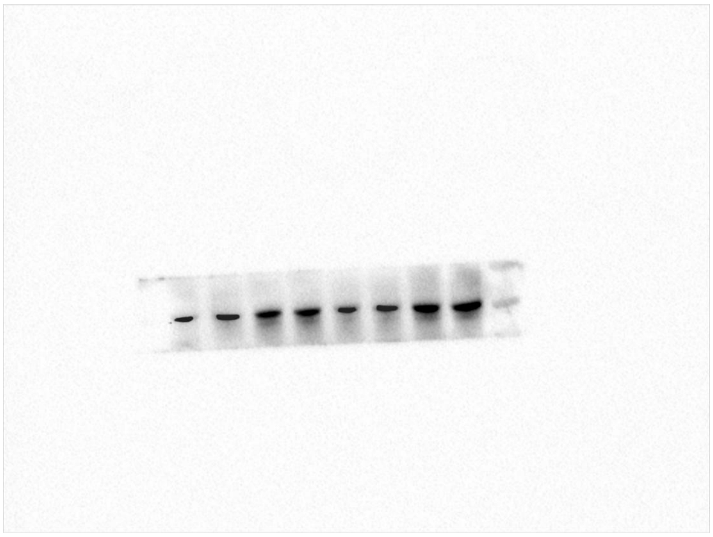

PI3K

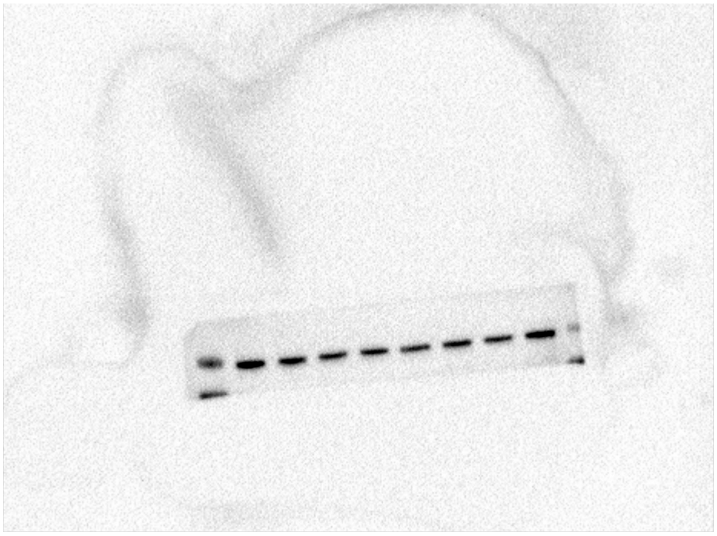

GAPDH

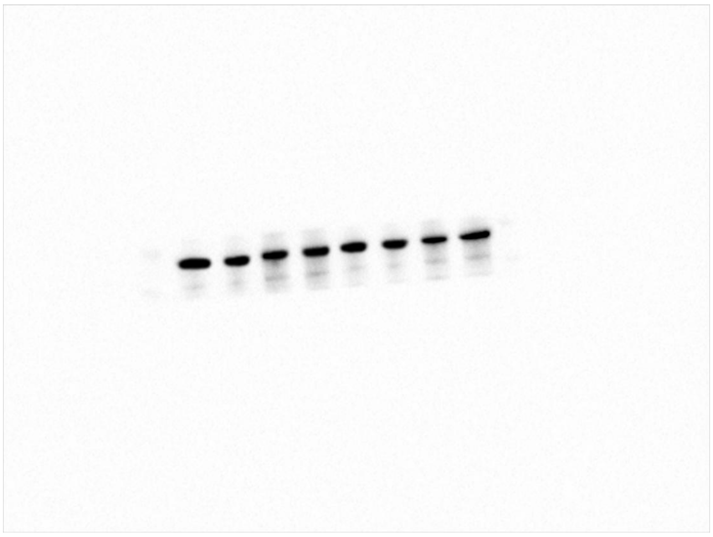

P-PI3K

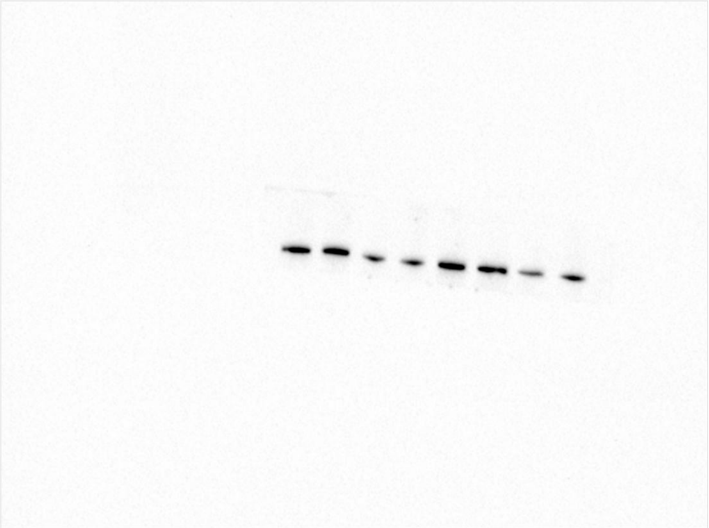

AKT

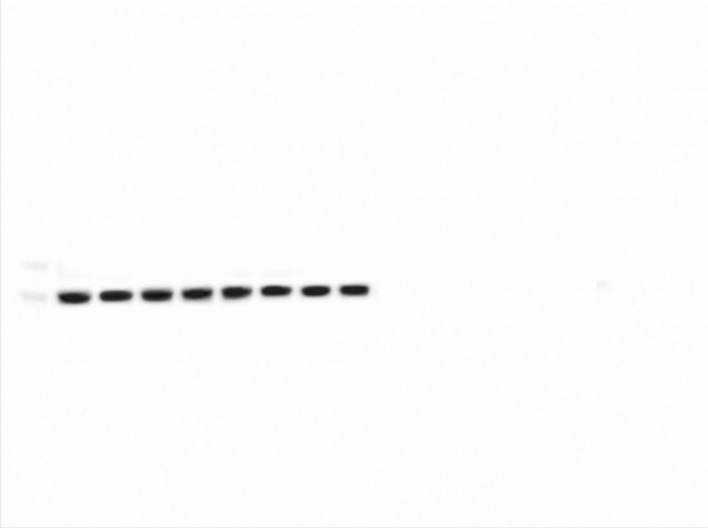

GAPDH

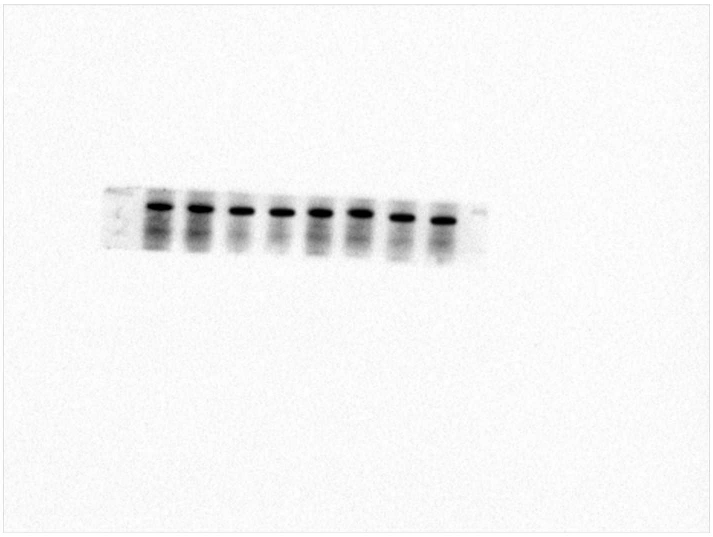

P-PI3K

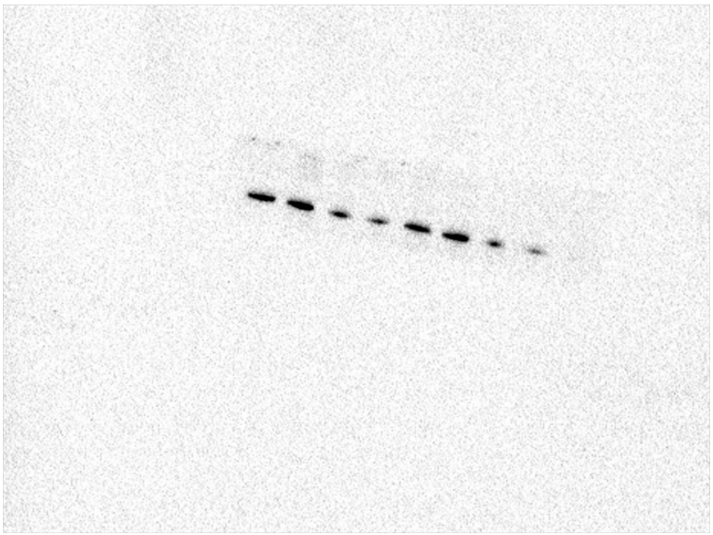

AKT

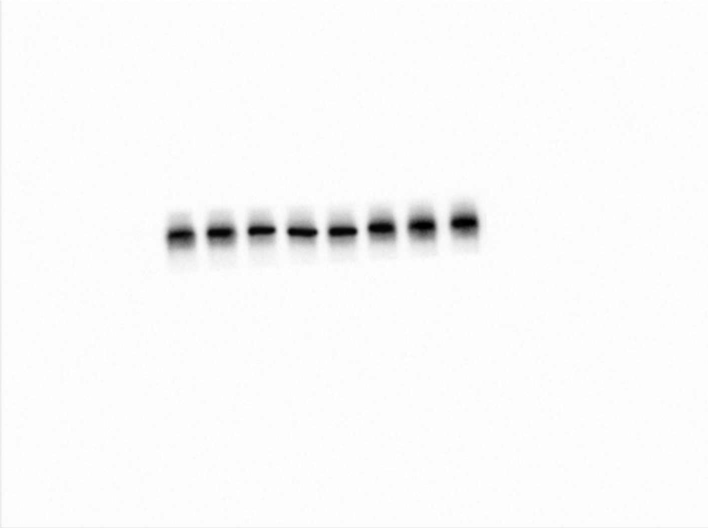

GAPDH

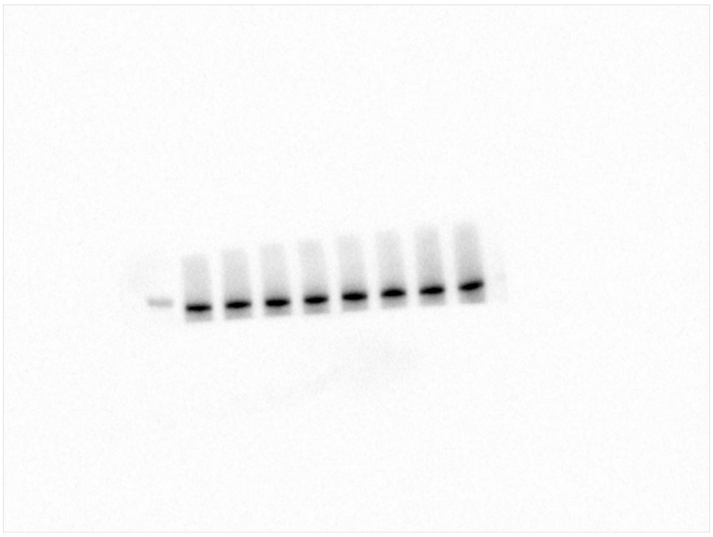

P-AKT

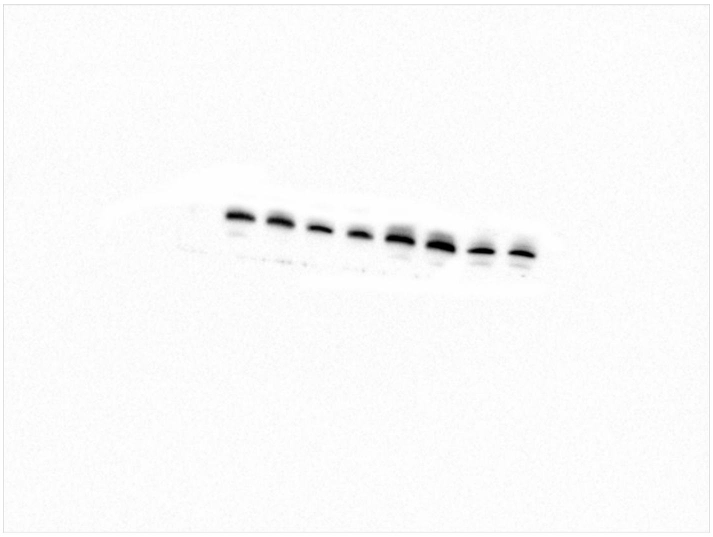

GAPDH

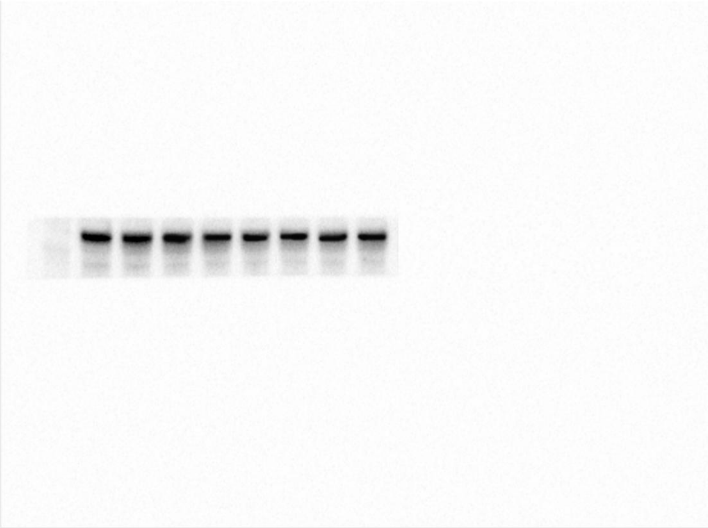

P-AKT

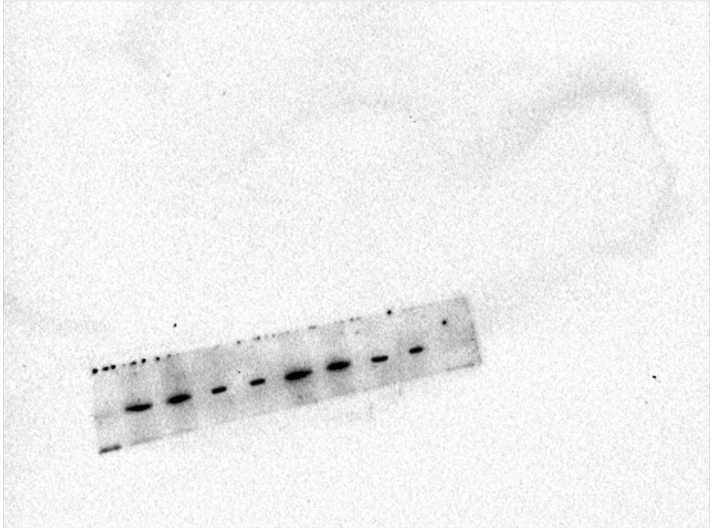

GAPDH

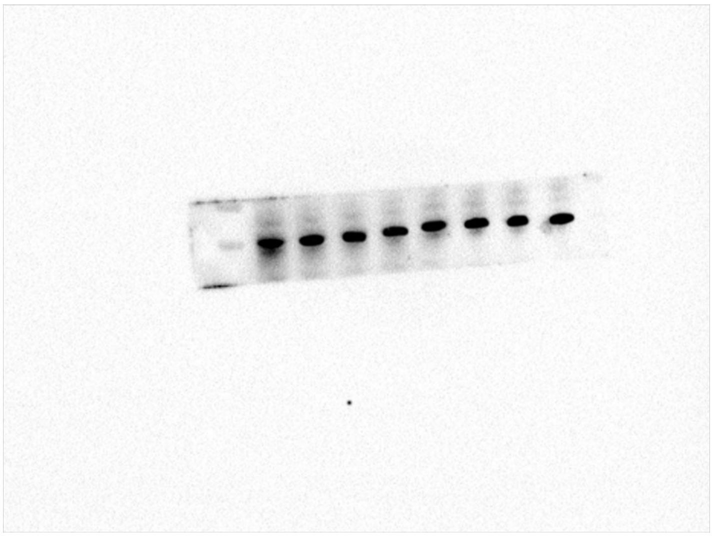

Supplement: Supplementary file 1 [file DataSheet1.pdf]
